# Bioinspired Microphase‐Engineered Binders for Silicon Anodes

**DOI:** 10.1002/advs.202522802

**Published:** 2026-02-21

**Authors:** Lirong Tang, Lan Zhao, Zhiyi Cao, Fengcai Lin, Biao Huang, Haijun Li, Lingling Qian, Yingshan Shi, Yaohang Weng, Xuan Yang, Hanyang Liu, Beili Lu, Jianhua Lv, Xinda You, Jiayu Tao, Zhenwei Wu

**Affiliations:** ^1^ College of Material Engineering Fujian Agriculture and Forestry University Fujian China; ^2^ Fujian Engineering and Research Center of New Chinese Lacquer Materials College of Materials and Chemical Engineering Minjiang University Fuzhou China; ^3^ School of New Energy Ningbo University of Technology Ningbo China

**Keywords:** bioinspired materials, mechanophores, microphase engineering, silicon anodes, solid electrolyte interphase

## Abstract

Silicon anodes require binders that not only buffer volume changes but also preserve interfacial integrity. However, excessive encapsulation limits ion transport and long–term stability. Here, we develop a lipoic acid–rosin acrylate (LRA) binder via thiol–ene click chemistry. LRA exhibits high stretchability (4154%) and self–assembles into chain motifs, forming microphase arrangements in aqueous media. Incorporated into hinged–tethering phosphorylated cellulose nanocrystals (HT–PCNCs) and ionically crosslinked alginate–Ba^2^^+^ scaffolds, these motifs cluster into mesoscale domains, reminiscent of the armor plates of *Phloeodes diabolicus*. This hybrid structure integrates rigid backbones with deformable rosin–rich beads, enabling localized strain dissipation, self–repair, and regulated ion conduction for stable solid electrolyte interphase (SEI) formation. The elastic mosaic dispersed within HT–PCNCs/SA–Ba^2^^+^ provides mechanical robustness and ionic accessibility. The composite binder achieves a tensile strength of 308.52 MPa, fracture energy of 3288.48 MJ m^−^^3^, and ionic conductivity of 33.607 mS cm^−^^1^, while effectively suppressing interfacial cracks. Silicon electrodes deliver 83.25% capacity retention after 100 cycles, high rate capability (869.8 mAh g^−^^1^ at 3C), and long‐term durability (1798 mAh g^−^^1^ after 300 cycles), accompanied by an ultrathin (∼17 nm) LiF–rich SEI. This work highlights spatially resolved microphase engineering as a promising strategy for adaptive bio–based binders in silicon anodes.

## Graphical abstract

Inspired by the tough exoskeleton of Phloeodes diabolicus, a bio‐based binder featuring LRA–click chemistry and hierarchical crosslinking forms well‐defined microphase‐separated structures that enable robust silicon anodes. This system promotes the formation of a LiF–rich, ultrathin SEI (∼17 nm) with high modulus and ionic conductivity, ensuring long‐cycle stability. This work demonstrates a bioinspired strategy for constructing mechanically and interfacially adaptive silicon‐based lithium‐ion batteries through microstructural network design.

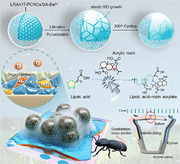

## 1 Introduction

Silicon (Si) has garnered significant attention as a next–generation anode material due to its ultrahigh theoretical capacity (4200 mAh g^−^^1^). However, its practical implementation is impeded by drastic volume expansion during lithiation/delithiation, which causes electrode pulverization, interfacial instability, and rapid capacity fade [1, 2]. To mitigate this, advanced polymeric binders engineered to buffer these fluctuations have emerged as a pivotal solution for stabilizing Si anodes [3, 4]. In recent years, the superior performance of silicon anodes with aqueous binders, traditionally attributed to hydrophilicity, primarily stems from the formation of dynamic 3D chain–particle networks [5]. These matrices dissipate stress from volume expansion through mechanical interlocking and swelling elasticity, enabling microscale interfacial restructuring that ensures long–term electrode integrity [6]. For instance, Hu et al. developed a multiscale rigid–flexible network using poly(acrylic acid) and poly(vinyl alcohol) for enhanced energy dissipation [7], while Choi et al. leveraged mechanically interlocked polyrotaxanes to mitigate stress [3]. Similarly, Cao et al. utilized catechol‐functionalized chitosan to balance stiffness with stress relaxation [8]. Despite simultaneous advances in conductivity [9], hierarchical structuring [10], and self‐healing [11], recent strategies have increasingly prioritized dense, continuous polymer networks to maximize particle encapsulation. While creating a uniform, compact coating can temporarily suppress particle detachment, this “the tighter, the better” paradigm imposes intrinsic limitations. Excessive encapsulation restricts ion transport pathways and hinders the renewal of interfacial chemistry, creating barriers to long–term electrochemical performance. Under rapid charge–discharge conditions, such rigid coverage is susceptible to causing localized ionic depletion or accumulation zones which promote irreversible thickening of the solid electrolyte interphase (SEI), thereby accelerating capacity loss over time [12, 13].

Nature offers sophisticated solutions to such mechanical contradictions. Biological structural materials often achieve exceptional mechanical performance by organizing soft and hard domains into finely tuned microphase–separated structures [14]. Among these, the diabolical ironclad beetle (*Phloeodes diabolicus*) exhibits extraordinary toughness, withstanding compressive loads nearly 39 000 times its body weight, through the synergistic arrangement of interdigitated medial sutures and layered microstructures [15]. Inspired by this mechanical interlocking of compliant and rigid domains, we propose a bio‐mimetic strategy, introducing spatially resolved microphase architectures into binder design to create “breathable” domains within a robust scaffold. This approach establishes a dual‐functional landscape where the rigid backbone ensures global mechanical integrity, while dispersed microphases integrate force–responsive mechanophores [16, 17, 18, 19] to facilitate localized stress dissipation and provide expedited channels for ion transport. Such structural adaptivity is essential for balancing modulus with flexibility and supporting continuous interfacial accommodation [20, 21].

**FIGURE 1:**
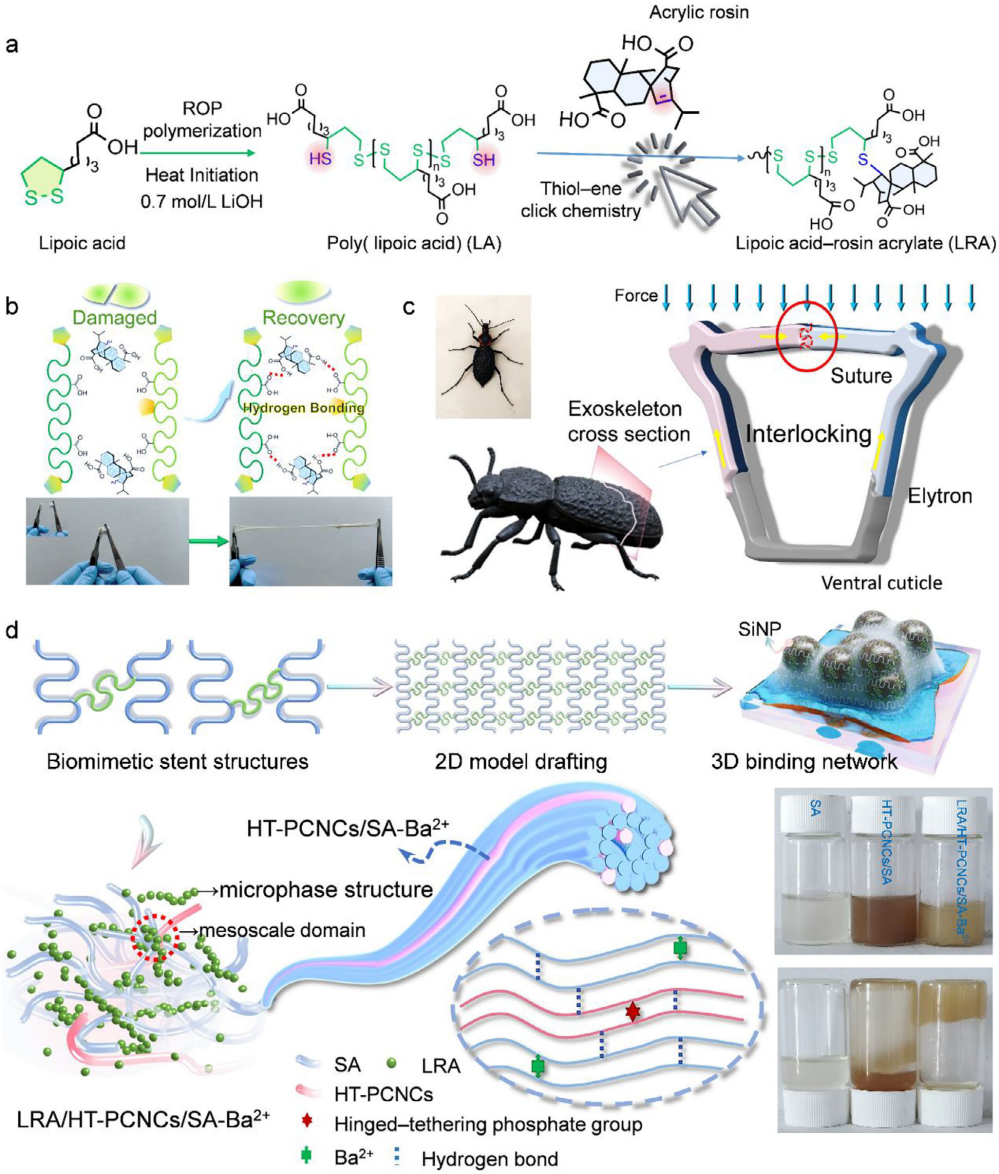
(a) LRA preparation flow chart. (b) Schematic diagram of the construction of the SEI layer in the silicon anode with LRA/HT–PCNCs/SA–Ba^2^^+^. (c) Schematic illustration of *Phloeodes diabolicus’* elytra structure. (d) Hierarchical network design process of the bionic flexible structure of LRA/HT–PCNCs/SA–Ba^2^^+^.

Lipoic acid (LA) integrates a dynamically reactive 1,2–dithiolane ring with a flexible octyl backbone. Its disulfide bond can efficiently anchor rigid unsaturated skeletons via thiol–ene click chemistry [22]. More critically, its endogenous C_8_ methylene chain acts as a “flexible tether”, effectively mitigating the steric hindrance of rigid side groups. This rigid–flexible coupling imparts superior segmental mobility to the oligomers, establishing the structural basis for self‐assembly into ordered microphases [23, 24]. Complementarily, Rosin, featuring a rigid tricyclic diterpene skeleton and conjugated double bonds, promotes interfacial π–π interactions and stabilizes SEI components [25, 26]. By clicking rosin onto the LA terminus, the resulting system exploits the thermodynamic incompatibility between the rigid hydrophobic moieties and the hydrophilic segments. Driven by polarity differences in alkaline aqueous media, these molecules spontaneously self‐assemble to construct intrinsic, highly elastic microphase structures, offering a potential reservoir for stress dissipation (Figure 1a) [27, 28].

In this study, we propose a strategy shifting from conventional dense encapsulation to adaptive microphase regulation for silicon anodes, as illustrated in Figure 1a,b. To effectively translate LRA microphases into a functional aqueous binder, we bridge the gap between their elasticity and the requirement for ionic transport by embedding them within an ionically coordinated alginate (SA–Ba^2^^+^) matrix reinforced by hinged–tethering phosphorylated cellulose nanocrystals (HT–PCNCs). Specifically, the SA–Ba^2^^+^ framework preserves continuous ionic pathways, while the HT–PCNCs provide the macroscopic modulus required to suppress electrode swelling, and the dispersed LRA domains function as localized stress–dissipation centers (Figure 1c,d). Mimicking the stiffness–toughness gradient from the exoskeletal of *Phloeodes diabolicus*, these adaptive microdomains effectively accommodates volume expansion while maintaining interfacial stability, providing a practical design for next–generation adaptive bio‐based binders of silicon anodes.

## 2 Results and Discussion

### 2.1 Synthesis and Characterization of Thiol–Ene Click Crosslinked Lipoic Acid–Rosin Polymer and the Composite Binder

**FIGURE 2:**
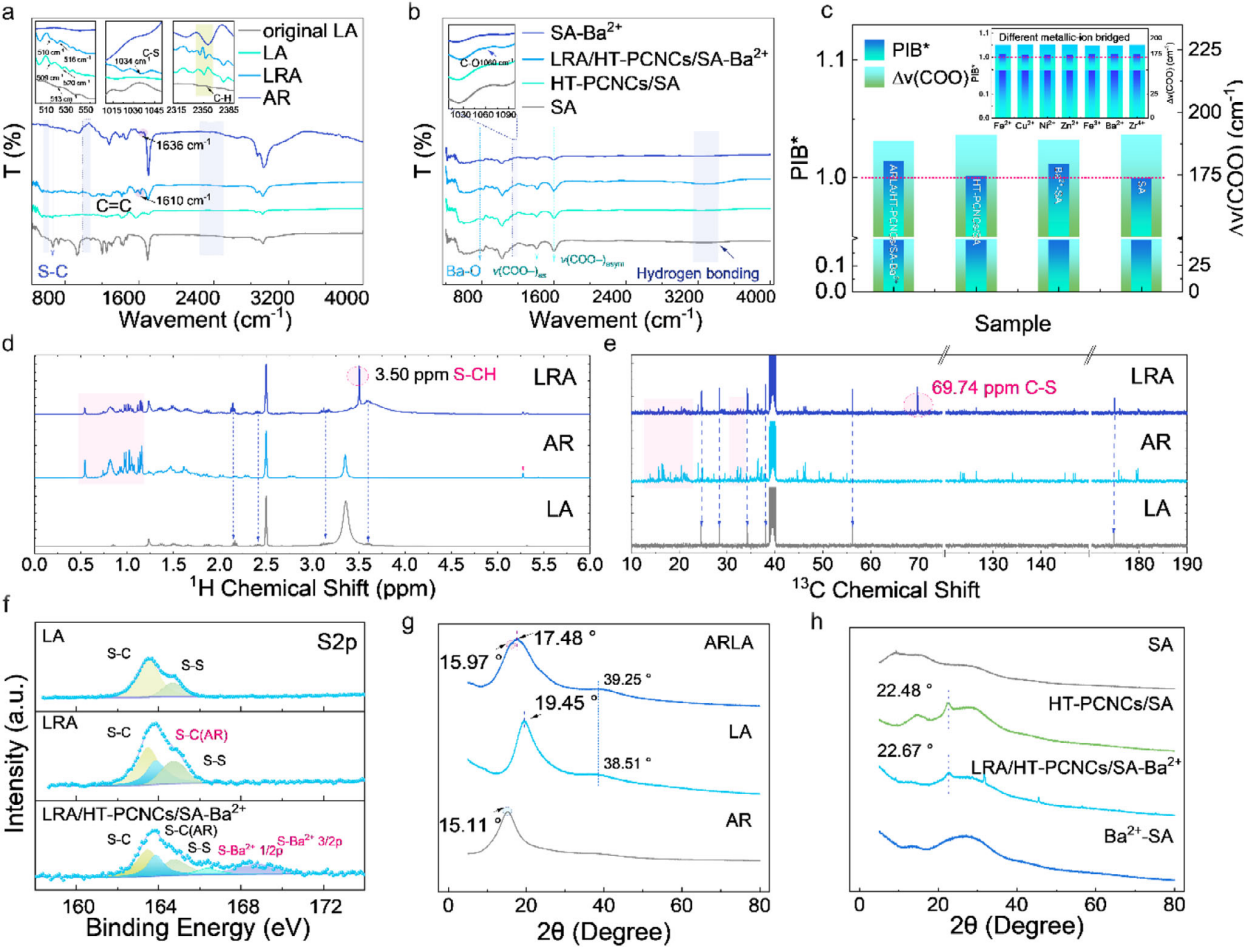
(a) FTIR of small molecular rosin‐based polymers and (b) binders. (c) PIB. (d) ^1^H NMR and (e) ^13^C NMR of small molecule rosin–based polymers. (f) XPS S 2p of small molecule rosin‐based polymer and binders. XRD of (g) small molecule rosin‐based polymer and (h) binders.

Under alkaline conditions, lipoic acid underwent thermally induced ring–closing polymerization, while acrylated rosin was introduced as a terminal radical quencher, resulting in the successful synthesis of a lipoic acid–rosin polymer (LRA). To elucidate the structural relationship between lipoic acid and acrylated rosin, the LRA was characterized by Fourier‐transform infrared spectroscopy (FTIR), proton nuclear magnetic resonance (^1^H NMR), and carbon–13 nuclear magnetic resonance (^1^ ^3^C NMR). In the FTIR spectrum (Figure 2a, b), new absorption peaks appeared in the 1036–1059 cm^−^^1^ region of the LRA, which were attributed to the formation of S─Ar bonds [29]. Additional peaks at 1616 and 1034 cm^−^^1^ likely originated from the conjugated groups of acrylated rosin and structures formed through thiol–ene click reactions with lipoic acid, indicating effective chemical linkage between the two components. Peaks observed at 510 and 516 cm^−^^1^ were attributed to S─S bonds; their shifts relative to pristine lipoic acid may reflect the influence of the conjugated rosin structure, suggesting a change in the molecular environment following polymerization [30]. As shown in Figure 2b, the peaks at 1597.5 and 1408.9 cm^−^^1^ corresponded to the asymmetric and symmetric stretching vibrations of carboxylate groups (─COO^−^), respectively [31]. The C─O stretching vibration observed at 1060 cm^−^^1^ indicated that further crosslinking may have occurred between hinged–tethering phosphorylated cellulose nanocrystals (HT–PCNCs) and LRA within the sodium alginate matrix.

Complementary evidence was obtained from the NMR spectra (Figure 2d,e). In the ^1^H NMR spectrum (Figure 2d), a new signal at 3.50 ppm was observed, corresponding to the S─CH bond formed in LRA, further confirming the occurrence of the click reaction. Meanwhile, a characteristic signal at 69.74 ppm in the ^1^ ^3^C NMR spectrum (Figure 2e) supported the formation of S─C bonds [23].

Furthermore, the chelation vibration peak of Ba–O confirmed that Ba^2^^+^ coordinated through multiple functional sites, such as S–H, C═O, and C–O, integrating into the crosslinked network and contributing to the stabilization of the composite structure [32]. As shown in Figure S1 ESI†, compared with Ba^2^^+^–SA, the incorporation of LRA resulted in a potentially denser crosslinked network in the LRA/HT–PCNCs/SA–Ba^2^^+^ system, as indicated by its higher polyion–binding index (PIB) value.

To assess the coordination strength between various metal ions and carboxyl groups in the binder network, the PIB was determined from FTIR spectra (Figure 2c). Introducing Ba^2^^+^ into the LRA/HT–PCNCs/SA matrix resulted in the highest PIB value (1.0137), notably exceeding those of Fe^3^^+^ and Zr^4^^+^ despite their higher charge. This suggests that Ba^2^^+^ may achieve more effective coordination within this multicomponent system. One likely explanation involves the quasi‐microphase‐separated architecture of LRA, which presents spatially distinct carboxyl‐rich and flexible domains at the nanoscale. The relatively large ionic radius and moderate charge density of Ba^2^^+^ could enable it to better access these segregated regions, facilitating uniform crosslinking across soft and hard segments within the hybrid network. By contrast, highly charged ions such as Zr^4^^+^ tend to trigger rapid localized aggregation rather than forming a continuous network. This heterogeneous precipitation restricts the accessibility of binding sites throughout the matrix, thereby reducing the overall integration, reflected in a lower PIB value, and potentially disrupting the ordered microphase organization [33]. These findings imply a unique structural compatibility between Ba^2^^+^ and microphase–separated LRA–based networks.

**FIGURE 3:**
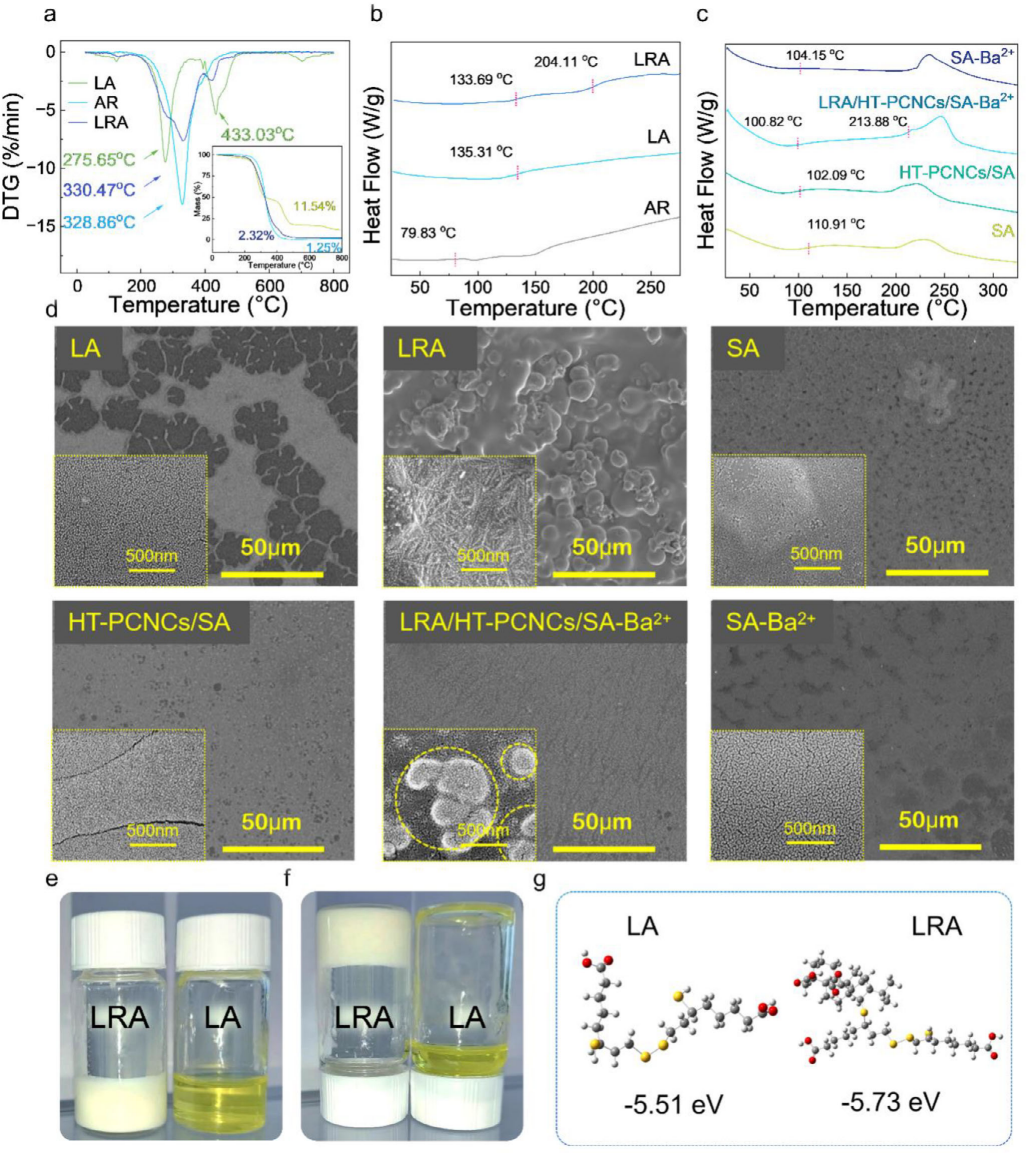
(a) TG and (b) DSC of small molecule rosin‐based polymer. (c) DSC of binders. (d) SEM image the network of binders. (e,f) Optical images of LA and LRA. (g) The binding energy between different polymer units.

In the X‐ray photoelectron spectroscopy (XPS) C 1s spectrum (Figure S2), a weak C═C peak at 283.1 eV and a significantly enhanced C─O/C─S peak at 286.4 eV indicated the formation of a stable covalently bonded composite structure between LRA and HT–PCNCs/SA, consistent with the FTIR results [34]. The S 2p spectrum (Figure 2f) further confirmed that the introduction of AR eliminated the thiol radicals present in lipoic acid and altered the sulfur bonding states, thereby promoting the formation of an ordered chain structure (Figure 3d; Figure S4). The S 2p spectra of LA and LRA were deconvoluted into distinct peaks. LA exhibited two major peaks corresponding to S─S bonds at 164.7 eV and C─S bonds at 163.5 eV. In contrast, the S 2p spectrum of LRA showed, in addition to the original S–S and C–S peaks, a new thioether peak at 163.8 eV, confirming chemical modification through thiol–ene click reaction [23]. Furthermore, LRA/HT–PCNCs/SA–Ba^2^^+^ formed notable S–Ba^2^^+^ coordination bonds, contributing to improved uniformity and structural integrity of the crosslinked network.

XRD analysis (Figure 2g) revealed that AR and LA exhibited characteristic diffraction peaks at 15.11° and 19.45°, respectively. In contrast, the LRA composite showed shifted peaks at 15.97° and 17.48°, indicating disrupted crystallinity due to molecular interactions. The shift and peak broadening suggest segmental rearrangement and the emergence of localized structural domains, indicative of a quasi–microphase–separated morphology. Both LRA and LA displayed broad peaks, confirming reduced internal order. In LRA, this may reflect the presence of localized structural heterogeneity, possibly linked to microphase‐like domain formation.

In the LRA/HT–PCNCs/SA–Ba^2^^+^ composite (Figure 2h), the (200) plane appeared more flattened at 22.48°, likely due to Ba^2^^+^–mediated bridging between LRA and the matrix. This further enhanced structural heterogeneity, extending the microphase characteristics to the composite scale.

For practical application as a battery binder, a material must not only exhibit high mechanical strength but also demonstrate excellent thermal stability and good electrolyte wettability to ensure enhanced composite performance. Upon incorporation of AR, the thermal degradation peak of LA shifted from 275.65°C to 330.47°C, representing an increase of nearly 20% (Figure 3a). When further combined with HT–PCNCs/SA, the degradation peak increased to 246.45°C, significantly higher than those of the other samples (Figure S3), indicating improved thermal resistance and network stability.

DSC analysis provides clear thermal evidence of microphase separation in the binder systems. LA and AR each show a single T_g_ (135.31°C and 79.83°C), indicating homogeneous networks. In contrast, LRA exhibits two T_g_ values (133.69°C and 204.11°C), corresponding to flexible and rigid domains, respectively, a clear indication of segmental microphase separation (Figure 3b). When embedded in the HT–PCNCs/SA–Ba^2^^+^ matrix, LRA retains dual T_g_ (100.82°C and 213.88°C), with shifts reflecting interfacial plasticization and ionic confinement (Figure 3c). The persistence of dual transitions confirms that the microphase–separated structure is preserved within the composite. Single T_g_ profiles in control samples further support this structural distinction.

In conjunction with SEM observations (Figure 3d; Figure S4), the imaging provides final morphological validation of microphase–separated structures. While LA and AR exhibit dispersed nanoscale particles, LRA assembles into chain‐like, cocooned nanostructures (∼14 nm), consistent with its dual T_g_ and broad XRD peaks, evidence of intrinsic microphase separation.

This phase–separation behavior extends into larger architectures when LRA is incorporated into the HT–PCNCs/SA–Ba^2^^+^ matrix. The resulting hybrid forms well defined cocoon–like aggregates (∼260 nm), suggesting that LRA retains its segmental self–assembly capability and guides mesoscale organization even within multicomponent networks. In contrast, control systems lacking LRA (SA, SA–Ba^2^^+^, HT–PCNCs/SA) display only irregular, island‐like morphologies with no evident domain structuring, underscoring the structural homogeneity of these matrices.

Notably, the distinct size and order of these aggregates, compared to the smaller or absent clusters in Fe^2^^+^, Fe^3^^+^, and other metal systems, correlate closely with their respective PIB values, reflecting differences in coordination strength and network propagation. Zr^4^^+^ induces localized surface over–aggregation (∼336 nm) rather than integrated domain formation, likely due to excessive charge density disrupting chain mobility. Taken together, the persistence of dual T_g_, XRD peak broadening, elevated PIB, and SEM morphologies in the Ba^2^^+^ system collectively confirm the successful construction of a microphase–separated, hierarchically organized polymer network.

### 2.2 Performance Evaluation of the Functionalized Composite Binder

**FIGURE 4:**
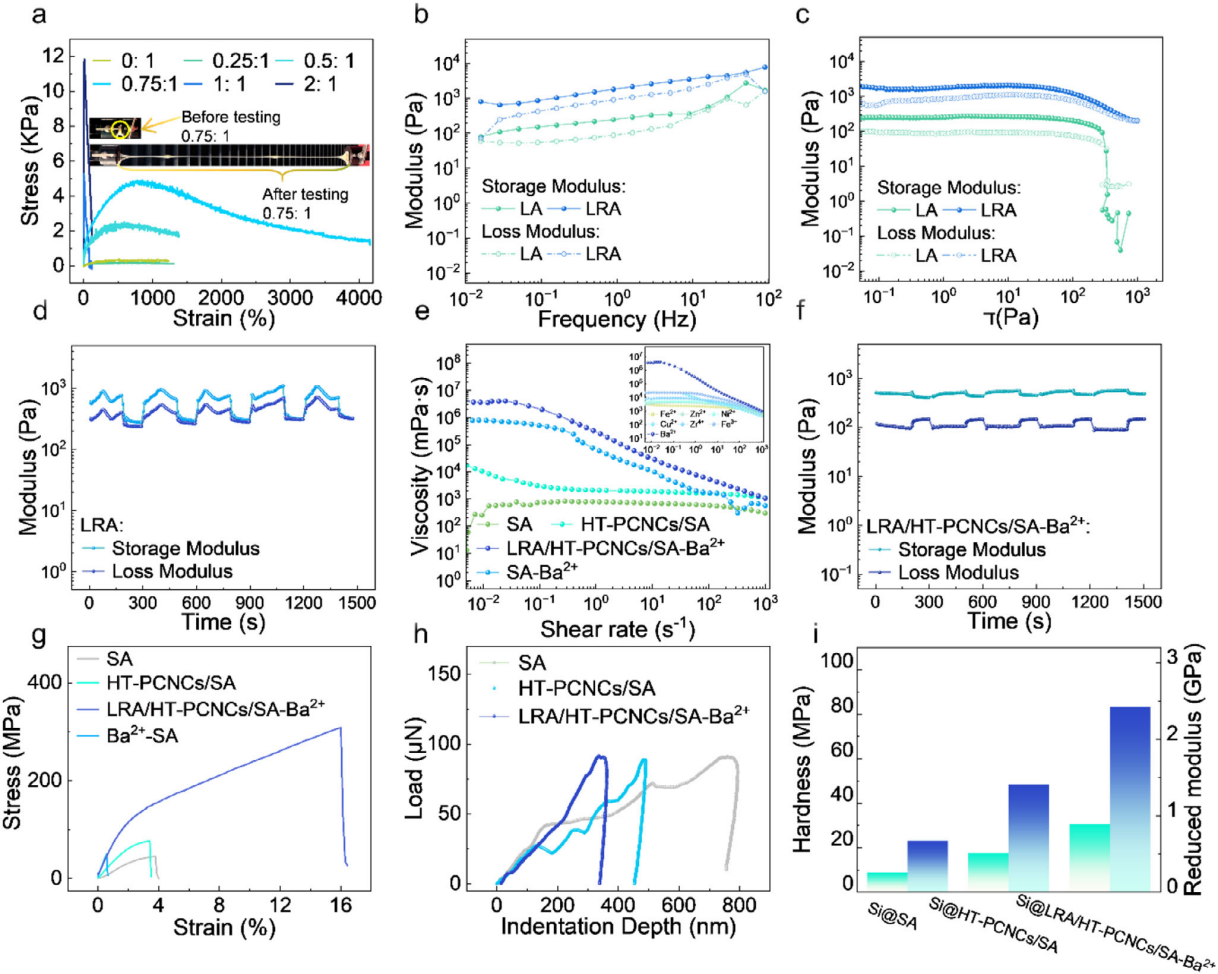
(a) Tensile properties, (b) dynamic frequency sweep, and (c) dynamic stress sweep of small molecule rosin‐based polymers. (d) Strain amplitude dynamic alternate sweep of LRA. (e) Rheological behavior of binder (5 wt.%). (f) Strain amplitude dynamic alternate sweep of LRA/HT–PCNCs/SA–Ba^2^^+^. (g) Tensile properties of binders. (h) Nanoindentation. (i) Hardness and modulus.

To accommodate the substantial volumetric fluctuations of silicon anodes during cycling, binder materials must exhibit both high tensile resilience and well‐tuned rheological properties, key performance targets in the design of LRA (Figure 3e,f). When synthesized at a molar ratio of acrylic rosin to lipoic acid of 0.75:1 (0.63 g:0.5 g), the resulting LRA demonstrated an exceptional elongation at break of 4154% (The tensile behavior of the LRA sample is shown in Video S1), representing a 337% increase over that of pure LA. Simultaneously, its tensile strength increased by 1746%, attributed to molecular reinforcement provided by the acrylic rosin, which functions as a dynamic network modulator (Figures 3g and 4a, binding energies: LA (−5.51 eV), LRA (−5.73 eV)).

Rheological profiling revealed that, although LRA and LA exhibited similar trends, the incorporation of AR resulted in more than an order of magnitude increase in viscosity (Figure S5), storage modulus (Figure 4b), and loss modulus (Figure 4c). Notably, LRA demonstrated superior elastic recovery and structural stability under strain (Figure 4d; Figure S6), highlighting its enhanced energy dissipation and self‐restoration capabilities, attributes critical for maintaining electrode integrity under repeated mechanical deformation. (Self‐healing behavior as shown in Video S2)

Given that battery binders typically exist as liquids or highly viscous fluids, conventional tensile‐compression testing is often inadequate. In contrast, rheological analysis provides a robust approach for simulating and characterizing the stress–strain behavior of liquid and semi‐solid materials, offering direct insights into their energy dissipation capacity [35]. As shown in Figure 4e, the incorporation of LRA enabled the binder system to retain high viscosity while exhibiting pronounced shear‐thinning behavior. This rheological signature ensures excellent slurry processability for the LRA/HT–PCNCs/SA–Ba^2^^+^ composite, promoting homogeneous dispersion and minimizing electrochemical performance fluctuations caused by compositional heterogeneity.

Dynamic frequency sweep tests (Figures S7 and S8) revealed that the LRA/HT–PCNCs/SA–Ba^2^^+^ system exhibited excellent viscoelastic tunability. The loss modulus (G'') consistently exceeded the storage modulus (G'), and both increased with frequency, indicating that the system predominantly dissipated energy in response to external deformation, an essential characteristic for maintaining rheological stability under dynamic conditions. This behavior is attributed to the microphase–separated architecture established by LRA within the crosslinked network, where reversible bonds and flexible domains enable continuous rearrangement of crosslinking sites under stress. Such a hierarchical and adaptive structure facilitates efficient energy dissipation and mechanical compliance throughout cycling. Similar viscoelastic trends were observed in other metal ion–crosslinked LRA/HT–PCNCs/SA systems.

Dynamic stress sweep measurements (Figures S9 and S10) further demonstrated that the LRA/HT–PCNCs/SA–Ba^2^^+^ system maintained a high storage modulus across a wide stress range (0.1–1000 Pa), outperforming other samples in terms of strain tolerance. This superior mechanical resilience is critical for accommodating the extreme ∼300% volume expansion of nanosilicon during electrochemical cycling.

Strain amplitude step–cycle testing (0.1% strain for 200 s followed by 30% strain for 100 s, repeated over five cycles) was conducted to evaluate the reversibility and energy dissipation capacity of the binder system under periodic deformation (Figure 4f; Figure S11). This protocol simulates alternating low strain (elastic recovery) and high strain (intense shear) conditions, enabling the assessment of dynamic evolution in storage modulus (G′) and loss modulus (G″) under varying stress states. The LRA–embedded network structure facilitated rapid molecular–level reorganization, allowing the system to maintain rheological stability and recover its structural integrity even after exposure to 30% strain. In contrast, the SA–Ba^2^^+^ system exhibited weaker self‐healing behavior, with pronounced fluctuations in G′ and G″ during cycling, reflecting its limited adaptive capability. These findings are consistent with the stress sweep results, underscoring the critical role of LRA's dynamic crosslinking in promoting reversible network reconstruction and enhancing long–term mechanical resilience under cyclic strain [36].

From a macroscopic mechanical perspective (Figure 4g), the pristine SA material exhibited poor tensile performance due to its low crosslinking density. It demonstrated a tensile strength of only 45.32 MPa, an elongation at break of 3.78%, and a fracture energy of 104.51 MJ m^−^^3^. These results reflect a tendency toward brittle failure under high strain, with limited energy dissipation capacity. The incorporation of HT–PCNCs enhanced the tensile strength to 77.16 MPa. Notably, with the further introduction of LRA and Ba^2^^+^ coordination, the composite achieved significantly improved mechanical performance, including a fracture energy of 3288.48 MJ m^−^^3^, a tensile strength of 308.52 MPa, and an elongation at break of 16.01%.

In comparison, systems crosslinked with other metal ions (Figure S12), such as Ni^2^^+^ (7.26%, 73.48 MPa, 636.58 MJ m^−^^3^), Fe^2^^+^ (6.45%, 73.48 MPa, 670.81 MJ m^−^^3^), and Zn^2^^+^ (2.11%, 66.66 MPa, 224.31 MJ m^−^^3^), exhibited relatively high tensile strength but significantly lower fracture energies and deformability. This reduction in mechanical adaptability is attributed to the smaller ionic radii of these metal ions, which form more rigid coordination networks with limited strain accommodation capacity. These findings are consistent with the rheological profiles (Figure 4e; Figures S7–S10), confirming that the dynamic flexibility imparted by the LRA/Ba^2^^+^ network is essential for effective stress adaptation and mechanical durability.

Peel strength and nanoindentation tests are essential for quantitatively evaluating the mechanical behavior of silicon‐based anodes. These techniques assess not only the interfacial binding strength between binder molecular chains and active materials but also the system's adaptability and energy dissipation capacity under mechanical stress. As shown in Figure S13, the Si@HT–PCNCs/SA electrode exhibited a maximum peel strength of 2.98 N cm^−^^1^, slightly lower than that of pure SA, although the overall interfacial robustness was marginally improved. Upon incorporation of LRA, a significant enhancement was observed. Compared to the SA–Ba^2^^+^ system (0.56 N cm^−^^1^), the Si@LRA/HT–PCNCs/SA–Ba^2^^+^ composite electrode achieved a peel strength of 10.83 N cm^−^^1^, indicating a markedly strengthened binder–active material interface.

In nanoindentation tests (Figure 4h; i), different binder systems exhibited distinct mechanical responses under a 100 µN load. The Si@SA electrode showed a large indentation depth of 793.05 nm and a low recovery rate of 4.6%. The corresponding hardness and elastic modulus were 8.71 MPa and 0.67 GPa, respectively, indicating that SA alone provided insufficient mechanical constraint to suppress the volume expansion of nanosilicon, thereby increasing the risk of active material delamination during cycling. The incorporation of HT–PCNCs led to moderate mechanical improvement (deformation: 490.00 nm; recovery rate: 1.5%), although the effect remained limited. In contrast, the Si@LRA/HT–PCNCs/SA–Ba^2^^+^ system exhibited significantly enhanced performance, with deformation reduced to 248.70 nm, recovery rate increased to 6.4%, and hardness and elastic modulus elevated to 30.66 MPa and 2.42 GPa, respectively.

This enhancement is mainly due to the microphase–separated network formed by LRA, which provides robust interfacial adhesion and improved structural resistance to deformation. The reversible crosslinks enable effective stress distribution and energy dissipation, thereby promoting mechanical durability and long–term cycling stability of the electrode.

Beyond interfacial failure caused by insufficient mechanical strength, binder degradation during prolonged electrolyte immersion and high‐voltage cycling remains a critical challenge. Wettability tests revealed that the LRA/HT–PCNCs/SA–Ba^2^^+^ binder exhibited a contact angle of 24.124°, which further decreased to 17.718° after 10 s, significantly outperforming Si@SA, Si@LRA/HT–PCNCs/SA, and Si@Ba^2^^+^–SA in terms of electrolyte affinity (Figure S14). This superior wettability was also observed in comparison to binders cross‐linked with other metal ions (Figure S15).

After 540 min of electrolyte immersion, the swelling ratio of the LRA/HT–PCNCs/SA–Ba^2^^+^ film stabilized at 0.1422 ± 0.1371. Under the same conditions, the SA‐based film exhibited a swelling ratio of 0.6163 ± 0.2163, approximately 4.33 times higher (Figure S16). The large deviation arises from the low swelling level of the sample, where minor absolute changes cause notable relative variation. The incorporation of AR and HT–PCNCs effectively suppressed the intrinsic swelling tendency of SA in electrolyte environments. Although Ba^2^^+^ is traditionally considered unfavorable due to its relatively large ionic radius, in the context of the LRA/HT–PCNCs/SA system, this characteristic facilitated deeper embedding of LRA and HT–PCNCs. Consequently, it reinforced the overall network structure and mitigated electrolyte‐induced swelling. This performance advantage was clearly demonstrated in comparison with other control samples (Figure S17).

### 2.3 Electrochemical Performance of SiNP Anodes

**FIGURE 5:**
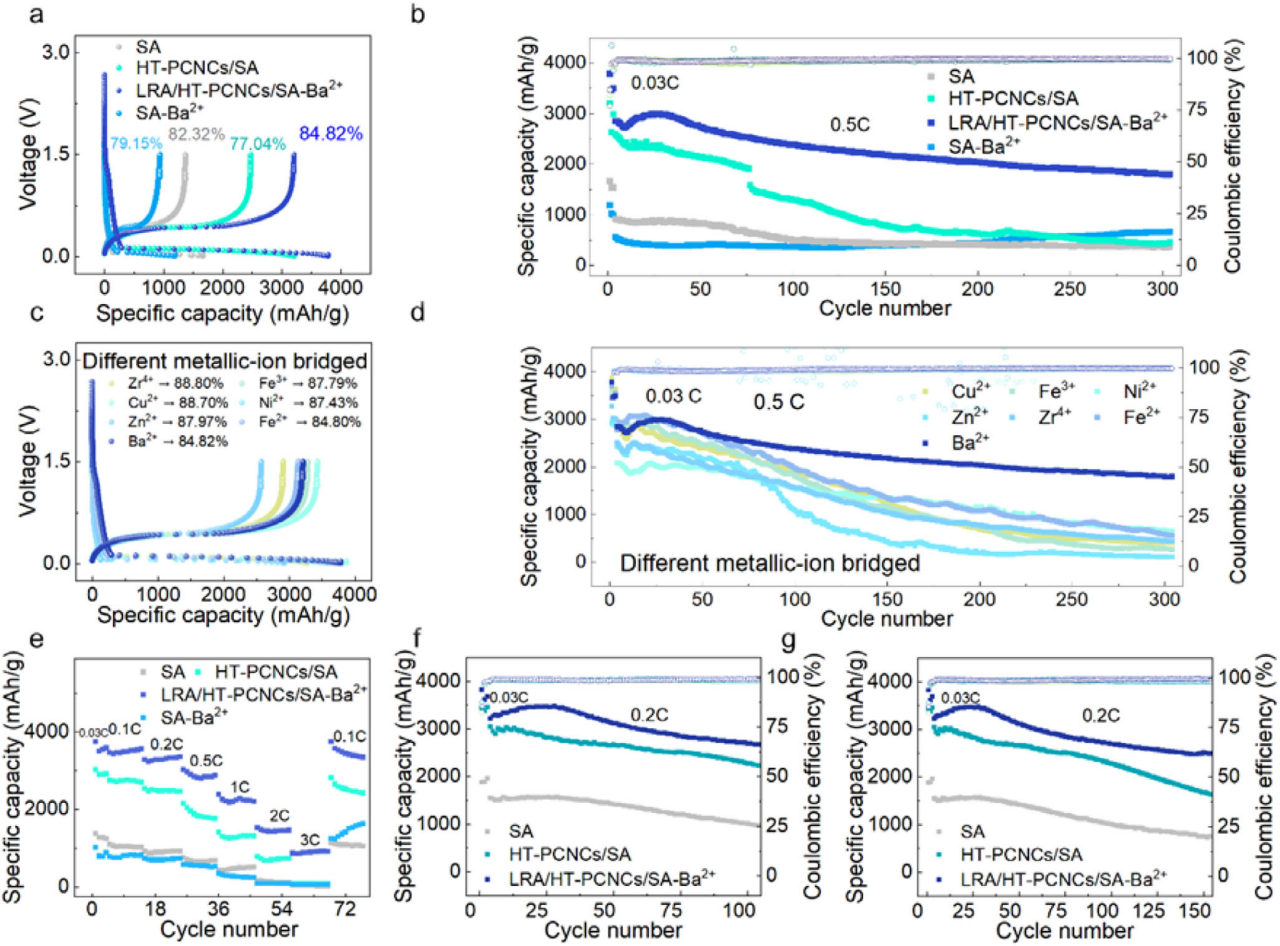
(a) Initial charge–discharge curves (0.03C), (b) cycling performance (0.5C) of Si electrodes. (c)Initial charge–discharge curves (0.03C), (d) cycling performance (0.5C) of Si electrodes with different metal ion cross‐linked binders. (e) Rate performance. Cycling performance at 0.2C for (f) 100 cycles and (g) 150 cycles.

The electrochemical performance of silicon nanoparticle (SiNP) anodes serves as a key indicator of their viability as high‐capacity anode materials. As shown in Figure 5a,b, the Si@LRA/HT–PCNCs/SA–Ba^2^^+^ electrode delivered an initial discharge capacity of 3783.9 mAh g^−^^1^ at a low current density of 0.03C. Even after 300 cycles at 0.5C, it retained a high reversible capacity of 1798.3 mAh g^−^^1^. This performance markedly outperformed that of control electrodes, including Si@SA (367.2 mAh g^−^^1^), Si@HT–PCNCs/SA (433.7 mAh g^−^^1^), and Si@Ba^2^^+^–SA (660.3 mAh g^−^^1^).

At a current density of 0.2C, the Si@LRA/HT–PCNCs/SA–Ba^2^^+^ electrode maintained a high specific capacity of 2695.2 mAh g^−^^1^ after 100 cycles, significantly outperforming both Si@SA and Si@HT–PCNCs/SA (Figure 5f). The rapid capacity degradation observed in the Si@SA electrode was primarily attributed to mechanical failure and the formation of an unstable SEI, resulting from the pronounced volume fluctuations of silicon during cycling. After 100 cycles, the expansion stress induced by repeated lithiation and delithiation increased the interfacial gaps between the binder and active particles. In the absence of a robust cross‐linked network, the binders, which depend primarily on hydrogen bonding, gradually lost their adhesion. Consequently, the HT–PCNCs/SA system also exhibited mechanical failure under sustained strain.

In contrast, the incorporation of LRA introduces microphase–separated domains within the composite binder network, effectively enhancing adaptability to volume changes and maintaining stronger interfacial contact with silicon particles. As a result, the Si@LRA/HT–PCNCs/SA–Ba^2^^+^ electrode retains a high specific capacity (2517.0 mAh g^−^^1^ after 150 cycles, Figure 5g) after prolonged cycling, demonstrating that such microstructural organization plays an important role in improving long–term stability of silicon‐based anodes.

In addition, the Si@LRA/HT–PCNCs/SA–Ba^2^^+^ electrode exhibited a favorable initial coulombic efficiency of 84.82% (Figure 5a,c). While displaying slight variations compared to other metal‐ion systems which may be attributed to the distinct electrolyte infiltration kinetics within the microphase domains, this result suggests that LRA/HT–PCNCs/SA–Ba^2^^+^ effectively stabilized the electrode–electrolyte interface during the initial activation process. As shown in Figure 5e, across a wide range of current densities (0.1C, 0.2C, 0.5C, 1C, 2C, and 3C), the Si@LRA/HT–PCNCs/SA–Ba^2^^+^ electrode consistently outperformed Si@SA, Si@HT–PCNCs/SA, and Si@Ba^2^^+^–SA. Its specific capacity decreased from 3466.6 mAh g^−^^1^ at 0.1C to 869.8 mAh g^−^^1^ at 3C. When the current density returned to 0.1C, the capacity recovered to 3580.0 mAh g^−^^1^, which was significantly higher than those of Si@HT–PCNCs/SA (1106.5 mAh g^−^^1^), Si@HT–PCNCs/SA (2652.9 mAh g^−^^1^), Si@Ba^2^^+^–SA (1245.1 mAh g^−^^1^), and Si@SA (1159.9 mAh g^−^^1^). Following the initial activation cycles, the full cells demonstrate stable operation at 0.5 C for over 50 cycles, delivering reversible discharge capacities of 84.7 mAh g^−^^1^ (LiFePO_4_) and 88.3 mAh g^−^^1^ (LiCoO_2_) at the end of cycling (Figure S18).

The outstanding rate and cycling performance stems from the synergistic integration of LRA, HT–PCNCs, and sodium alginate. Specifically, the Ba^2^^+^‐coordinated network outperforms other cation systems (Figure 5c,d). by establishing a mechanically robust yet flexible framework with superior fracture energy. This architecture simultaneously optimizes electrolyte wettability and ionic conductivity, ensuring sustained electrode integrity and rapid kinetics during cycling.

Cyclic voltammetry (CV) and electrochemical impedance spectroscopy (EIS) were conducted to further elucidate the electrochemical kinetics of the Si@LRA/HT–PCNCs/SA–Ba^2^^+^ electrode (Figure S19). Two anodic peaks at 0.39 and 0.53 V correspond to the delithiation of lithium–silicon alloys, consistent with the characteristic behavior of silicon‐based anodes [37]. Additionally, a cathodic peak at 0.17 V and the aforementioned anodic peaks closely match the lithiation and delithiation plateaus observed in the initial charge–discharge profiles. Notably, the gradual increase in redox peak intensity during cycling suggests enhanced electrochemical activation of the silicon anode Figure S19.

**FIGURE 6:**
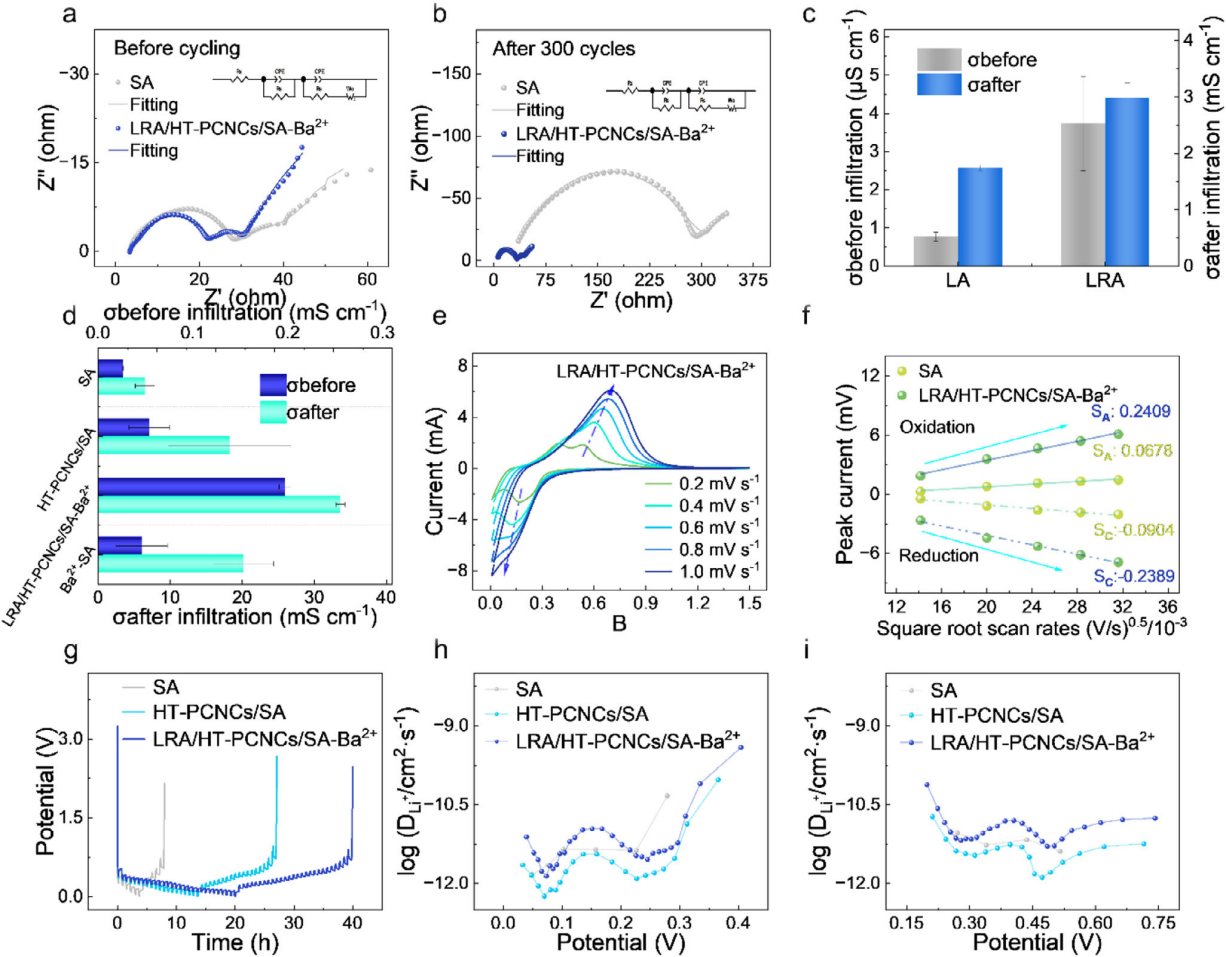
EIS curves of Si electrode (a) before cycling and (b) after 300 cycles at 0.5C. Ionic conductivity of (c) LA and LRA and (d) binders before and after electrolyte immersion. (e) CV curves of Si@LRA/HT–PCNCs/SA–Ba^2^^+^. (f) Relationship between peak current and the square root of scan rate. (g) GITT. Lithium–ion diffusion coefficient of Si@LRA/HT–PCNCs/SA–Ba^2^^+^ during (h) lithiumization and (i) delithiation.

Before cycling, the Si@LRA/HT–PCNCs/SA–Ba^2^^+^ electrode exhibits a low charge transfer resistance (R_CT_) of 4.82 Ω, significantly lower than that of the SA–based electrode (R_CT_ = 35.85 Ω). This indicates that the multidimensional network constructed by LRA and Ba^2^^+^ markedly enhances ionic transport and mechanical cohesion within the binder matrix. After 300 cycles at 0.5 C, EIS fitting reveals that the Si@LRA/HT–PCNCs/SA–Ba^2^^+^ electrode retains a relatively low interfacial resistance, with a solid electrolyte interphase resistance (R_SEI_) of 27.17 Ω and an R_CT_ of 25.14 Ω (Figure 6a,b). In comparison, the SA–based electrode displays substantially higher resistances, with R_SEI_ and R_CT_ reaching 284.60 Ω and 186.10 Ω, respectively. These findings underscore the superior interfacial stability and long‐term electrochemical kinetics afforded by the LRA/HT–PCNCs/SA–Ba^2^^+^ binder system.

In the dry state, all samples showed low conductivity due to limited electronic transport. As shown in Figure 6c, LRA improved conductivity over LA by 483.98% (3.730 ± 1.230 µS cm^−^^1^) before and 171.52% (2.989 ± 0.260 mS cm^−^^1^) after electrolyte infiltration. Incorporating LRA into the HT–PCNCs/SA matrix yielded 0.196 ± 0.006 mS cm^−^^1^ (Figure 6d), significantly higher than SA (0.026 ± 0.001 mS cm^−^^1^).

After infiltration, ion conduction dominated, and conductivity rose sharply [1, 6]. LRA/HT–PCNCs/SA–Ba^2^^+^ reached 33.607 ± 0.625 mS cm^−^^1^, outperforming all others. This is attributed to rosin–enhanced ion transport and balanced electrolyte uptake via metal coordination [28]. By contrast, SA (6.518 ± 1.344 mS cm^−^^1^) and Ba^2^^+^–SA (20.190 ± 4.187 mS cm^−^^1^) showed lower performance due to poor mobility or limited pathways. These results confirm that the microphase structure established by LRA‐based crosslinked networks creates interconnected ionic pathways while maintaining mechanical robustness, thus synergistically enhancing both conductivity and electrode stability for energy storage applications. Further comparison among the metal ion–bridged systems show that, except for Fe^3^^+^, all LRA–enabled networks outperform HT–PCNCs/SA, with the Ba^2^^+^– coordinated system consistently exhibiting superior ionic conductivity before and after electrolyte uptake (Figure S20).

### 2.4 Influence of Different Binders on the Reaction Kinetics of SiNP Anodes

CV measurements at varying scan rates and electrochemical stability tests were employed to evaluate the lithium‐ion diffusion coefficients (D_Li_^+^) of different binder systems. The Si@SA electrode exhibited a D_Li_^+^ range of 9.42 × 10^−^^1^ ^0^ to 1.67 × 10^−^^9^ cm^2^ s^−^^1^, whereas the Si@LRA/HT–PCNCs/SA–Ba^2^^+^ electrode demonstrated significantly enhanced values, ranging from 1.17 × 10^−^^8^ to 1.19 × 10^−^^8^ cm^2^ s^−^^1^ (Figure 6e,f; Figures S21 and S22). These results confirm that the LRA/HT–PCNCs/SA–Ba^2^^+^ binder system markedly improves lithium–ion transport while maintaining excellent stability during both lithiation and delithiation processes.

To further evaluate lithium‐ion transport kinetics under different states of charge and discharge, the galvanostatic intermittent titration technique (GITT) was employed. Based on Fick's second law [38], the D_Li_^+^ values of the Si@LRA/HT–PCNCs/SA–Ba^2+^ electrode remained consistently higher than those of the Si@SA electrode throughout the cycling process (Figure 6g–i). These results confirm that the synergistic integration of LRA and phosphorylated cellulose nanocrystals effectively mitigates silicon volume expansion and enhances structural stability during repeated cycling, thereby contributing to the superior electrochemical performance of the composite electrode.

### 2.5 Structural and Interfacial Characterization of SiNP Anodes

To gain deeper insight into the role of the composite binder in mitigating electrode volume expansion, SEM was employed to systematically investigate the microstructural evolution of electrodes during long–term cycling. In the pristine state, both Si@SA and Si@HT–PCNCs/SA electrodes exhibited smooth, crack‐free surfaces (Figure S23). However, after 300 cycles at 0.5C, pronounced surface cracks ranging from 1 to 17 µm in width appeared, indicating significant structural degradation.

**FIGURE 7:**
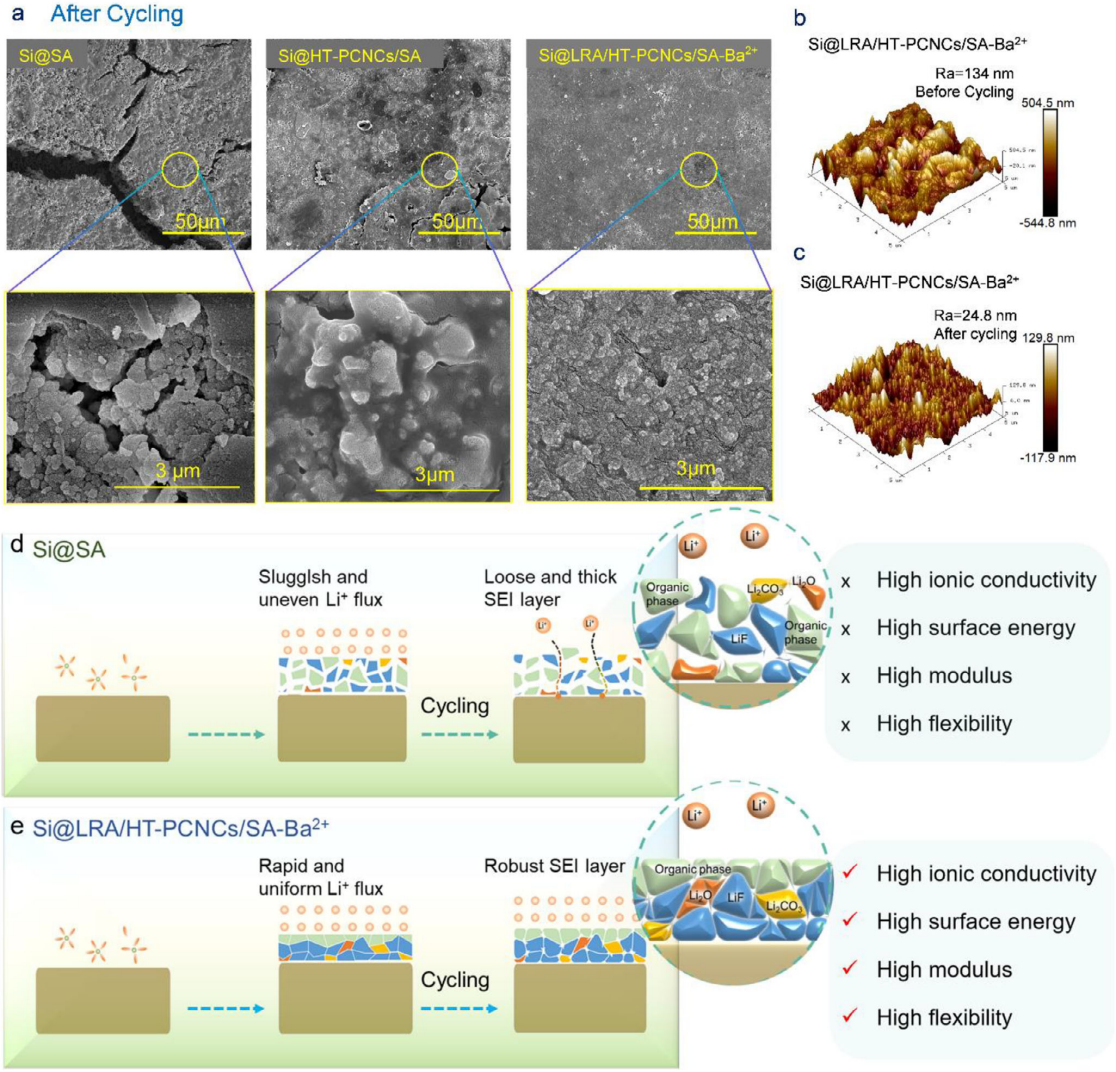
(a) Top view SEM images of electrodes after 300 cycles. AFM 3D images of Si@LRA/HT–PCNCs/SA–Ba^2^^+^ electrode (b) before and (c) after cycling. Schematic diagram of SEI layer deposition behavior with (d) SA and (e) LRA/HT–PCNCs/SA–Ba^2^^+^.

In contrast, the Si@LRA/HT–PCNCs/SA–Ba^2^^+^ electrode retained much greater structural integrity, exhibiting a denser surface and substantially fewer cracks after cycling (Figure 7a). This improvement was attributed to the energy‐dissipating capacity of the LRA/HT–PCNCs/SA–Ba^2^^+^ composite binder. Notably, the Si@HT–PCNCs/SA electrode developed large cracks (1–3 µm wide) that exposed fresh surfaces, disrupted the conductive network, and led to active material loss, ultimately resulting in severe performance degradation.

At higher magnification (3 µm scale), the Si@LRA/HT–PCNCs/SA–Ba^2^^+^ electrode exhibited markedly reduced expansion of the active material compared to the other binder systems after 300 cycles. Additionally, the binder remained uniformly distributed across the electrode surface, effectively encapsulating the silicon nanoparticles and contributing to improved mechanical and electrochemical stability.

In contrast, the large surface cracks observed on the Si@SA electrode further compromised its structural integrity and led to an increase in internal resistance. Conversely, the Si@LRA/HT–PCNCs/SA–Ba^2^^+^ electrode exhibited significantly lower internal resistance after cycling (Figure 6a,b), indicating that the LRA/HT–PCNCs/SA–Ba^2^^+^ binder effectively maintained structural integrity and the continuity of the conductive network. This, in turn, contributed to enhanced cycling stability and improved overall electrochemical performance.

The ability of the LRA/HT–PCNCs/SA–Ba^2^^+^ system to effectively buffer the volume changes of silicon particles during repeated lithiation/delithiation was likely attributed to the optimized physicochemical properties introduced by the synergistic effects of LRA and phosphorylated cellulose nanocrystals. This buffering capability played a crucial role in preserving electrode integrity and supporting long–term performance.

Further insights into the structural evolution of the composite electrodes were obtained by analyzing their thickness changes before and after 300 cycles using cross–sectional field–emission scanning electron microscopy (Figures S24 and S25). Initially, the Si@SA and Si@HT–PCNCs/SA electrodes exhibited thicknesses of 17.79 and 21.61 µm, respectively. After prolonged cycling, their thicknesses increased significantly, corresponding to expansion ratios of 56.94% and 17.26%, respectively. In contrast, the Si@LRA/HT–PCNCs/SA–Ba^2^^+^ electrode showed the smallest increase in thickness, from 19.07 to 21.31 µm, with an expansion ratio of only 11.70%.

Atomic force microscopy (AFM) was employed to monitor surface morphology changes during cycling (Figure 7b,c; Figure S26–S29). Compared to the Si@SA electrode, the Si@LRA/HT–PCNCs/SA–Ba^2^^+^ electrode exhibited a noticeably smoother surface after 300 cycles. Although the initial surface morphologies of all electrodes were similar, the Si@SA and Si@HT–PCNCs/SA electrodes developed significantly increased surface roughness, measuring 224 and 137 nm, respectively, after extended cycling, attributed to the severe volume fluctuations of SiNPs. In contrast, the Si@LRA/HT–PCNCs/SA–Ba^2^^+^ electrode maintained a much lower surface roughness of 24.8 nm, indicating enhanced structural stability.

The minimal volume expansion and absence of observable cracks in the Si@LRA/HT–PCNCs/SA–Ba^2^^+^ electrode indicate superior mechanical stability. This can be ascribed to the microphase–separated, elastic network established by LRA within the composite binder, which provides adaptive stress dissipation and efficient self‐healing capability. Such a hierarchical structure accommodates the substantial volume fluctuations of SiNPs during cycling, thereby maintaining electrode integrity and interfacial stability over prolonged operation (Figure 7d,e).

**FIGURE 8:**
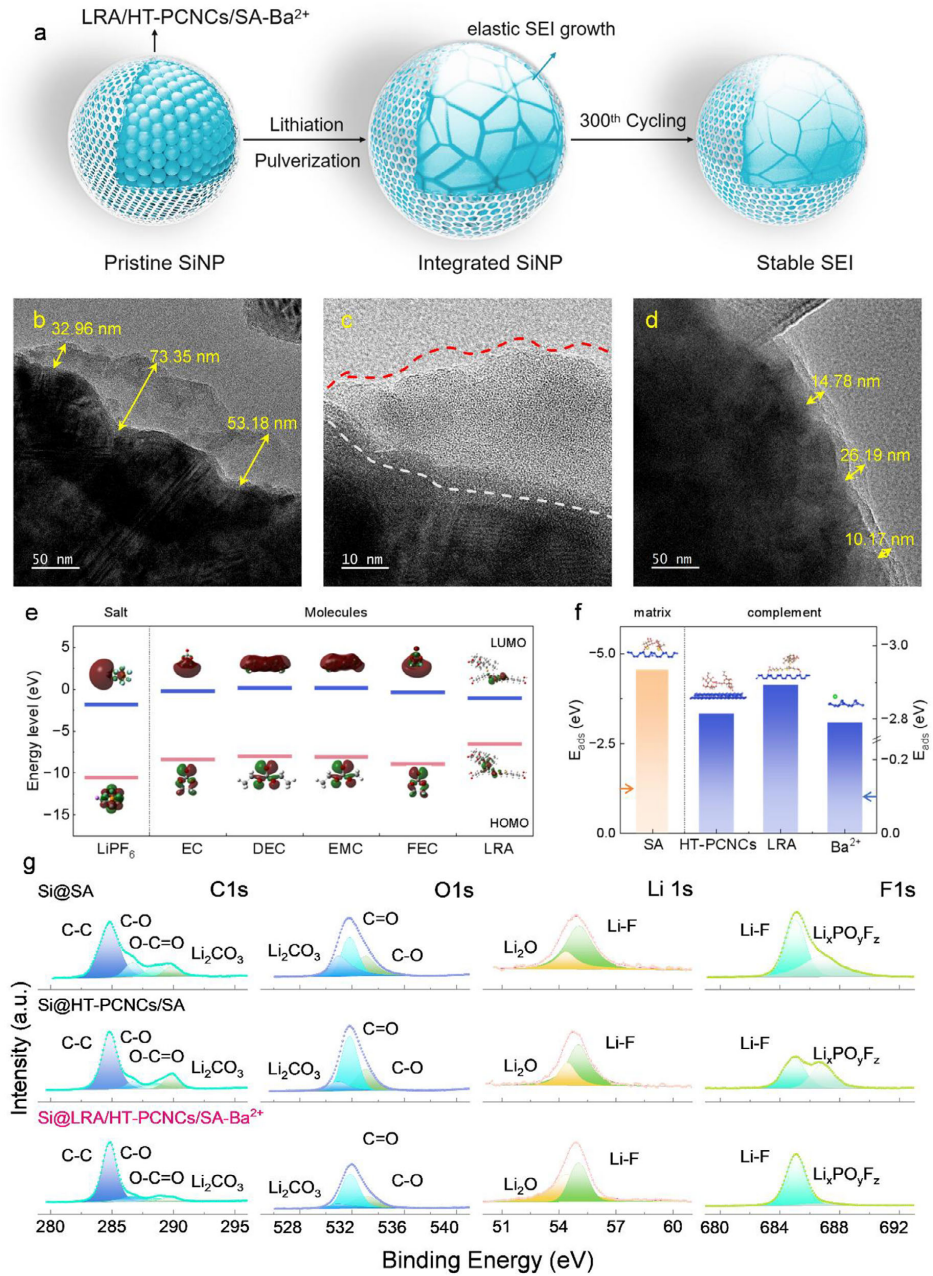
(a) Construction of SEI layer in silicon anode with LRA/HT–PCNCs/SA–Ba^2^^+^. HRTEM images of the Si@SA electrode at (b) 50 nm and (c) 10 nm after cycling and the Si@LRA/HT–PCNCs/SA–Ba^2^^+^ electrode at (d) 50 nm. (e) HOMO–LUMO simulation of different components. (f) Adsorption energy of SA, HT–PCNCs, LRA, and Ba^2^^+^. (g) The calculated frontier molecular orbitals and the optimized structures of molecules and molecular complexes.

High–resolution transmission electron microscopy (HRTEM) revealed that the Si@LRA/HT–PCNCs/SA–Ba^2^^+^ electrode exhibited a structurally intact and uniformly thick ultrathin SEI layer on the particle surface (Figure 8d), with an average thickness of approximately 17 nm. This SEI layer was continuous, compact, and conformal. In contrast, the SEI layer on the Si@SA sample was considerably thicker (∼53 nm), with a rough and fractured interface (Figure 8b,c). These structural defects led to repeated exposure of the silicon surface to the electrolyte, promoting persistent side reactions and uncontrolled SEI growth.

As illustrated in Figure S30, the LRA/HT–PCNCs/SA–Ba^2^^+^ binder facilitated the formation of a bilayer SEI structure, consisting of an inorganic–rich inner layer and an organic–rich outer layer, which effectively suppressed uncontrolled SEI thickening. The inner SEI primarily comprised stable inorganic species such as LiF, Li_2_O, and Li_2_CO_3_. In regions I, III, and VI, well–aligned LiF nanocrystals were observed, with lattice fringes corresponding to the (111) and (200) planes (0.232 and 0.201 nm, respectively), indicating that LiF was a dominant and structurally ordered component of the SEI. In regions II and V, lattice spacings of 0.163 and 0.267 nm were identified, corresponding to the (200) and (111) planes of Li_2_O, respectively. Additionally, region IV exhibited 0.211 nm lattice fringes consistent with the (104) plane of Li_2_CO_3_. These results confirmed the uniform distribution and structural integrity of the inorganic‐rich SEI layer [39, 40].

Furthermore, density functional theory (DFT) calculations were conducted to elucidate the reaction mechanisms involving LRA in lithium‐ion batteries. As shown in Figure 8e, LRA exhibited a LUMO energy level located strategically between the lithium salt (LiPF_6_) and carbonate solvents, indicating sufficient electrochemical stability to function as a functional binder.0020This property allowed LRA to act as an interfacial modulator that utilizes electrostatic attraction to anchor the decomposition products of LiPF_6_, thereby guiding the uniform deposition of inorganic species and promoting the formation of a LiF–rich SEI. Adsorption energy calculations (Figure 8f) showed that LRA had a stronger binding affinity to the Si surface compared to other binder components. This enhanced interfacial stability and promoted the formation of a robust, LiF‐enriched SEI structure.

To further investigate the chemical composition of the SEI, X‐ray photoelectron spectroscopy (XPS) was performed on cycled electrodes with different binders (Figure 8g). All three samples exhibited similar peaks in the C 1s spectra, with the dominant peak at 284.8 eV corresponding to C─C and C─H bonds from conductive carbon and organic SEI components. However, the Si@SA electrode showed a higher concentration of organic decomposition products, such as Li_2_CO_3_, alkyl carbonates, and alkyl carboxylates, compared to the Si@LRA/HT–PCNCs/SA–Ba^2^^+^ electrode, indicating more extensive electrolyte degradation [41].

In the O 1s and Li 1s spectra, the Si@LRA/HT–PCNCs/SA–Ba^2^^+^ electrode exhibited a more pronounced Li_2_O peak near the Si surface [42], whereas this peak was relatively weak in the Si@SA electrode. This observation suggests that the SA binder led to a thicker and less stable SEI, while the LRA‐based composite binder promoted the formation of a thinner, more stable, and Li_2_O‐enriched interphase.

Additionally, the F 1s spectra revealed notable differences in the contents of LiF and Li_x_PF_y_O_z_ among the three electrodes. The LiF peak at 685.0 eV was primarily attributed to the decomposition of lithium hexafluorophosphate (LiPF_6_, 686.7 eV) and fluoroethylene carbonate (FEC). When the SEI on silicon anodes is rich in LiF, the resulting passivation layer can exhibit lower resistance to Li^+^ diffusion, thereby enhancing cycling performance [43, 44]. Furthermore, the presence of Li_x_PF_y_O_z_ originated from the decomposition and electrochemical reduction of carbonate‐based electrolytes and lithium salt [45].

Changes in SEI composition are, in fact, largely governed by the physical properties of the binder [46]. When the binder exhibits weak hydrogen‐bonding interactions with silicon nanoparticles or possesses low mechanical strength, it tends to promote the formation of thick and fragile SEI layers on the silicon anode. This structural instability results in repeated exposure of fresh Si surfaces due to SEI fracture and decomposition, subsequently leading to the generation of Li_x_PF_y_O_z_ species [47].

Compared to the SEI formed on silicon anodes with pure SA binders, the formation of LiF and Li_x_PF_y_O_z_ was overall reduced in the SEI of Si anodes with HT–PCNCs/SA binders. Notably, the SEI formed on Si anodes using the LRA/HT–PCNCs/SA–Ba^2^^+^ binder was primarily composed of LiF. This was attributed to the enhanced mechanical strength of the binder and its strong hydrogen‐bonding interactions with Si nanoparticles, which effectively suppressed nanoparticle pulverization. As a result, a thin and stable SEI layer was formed, and electrolyte decomposition was mitigated (Figure 7d,e) [43].

Overall, the silicon anode with the LRA–containing binder maintained structural integrity during cycling, which facilitated the formation of a thin, LiF‐rich, and stable SEI layer (Figure 8a). This contributed to improved electrochemical performance and enhanced ionic conductivity [47, 48, 49].

## 3 Conclusion

Inspired by the rigid–flexible integrated exoskeletal structure of *Phloeodes diabolicus*, this study developed a supramolecular engineered, adaptive binder system based on highly elastic LRA to address the coupled mechanical and interfacial challenges of high–capacity silicon anodes. Distinct from conventional binders that form dense and continuous polymer matrices, our approach employs resolved microphase engineering to establish spatially organized domains with tunable elasticity and functional nanoscopic voids. Dynamic thiol–ene click chemistry enables reversible crosslinking within these domains, while mechanoresponsive disulfide bonds provide molecular‐scale energy dissipation under stress. The rigid tricyclic rosin backbone supplies essential mechanical reinforcement for structural stability during repeated cycling. To further enhance performance, we incorporated HT–PCNCs as reinforcing elements and established a sodium SA–Ba^2^^+^ ionic network for additional 3D support and improved ionic conductivity. This hierarchical composite architecture achieves both robust mechanical integrity and efficient ion transport pathways, demonstrated by its high tensile strength of 308.52 MPa, exceptional fracture energy of 3288.48 MJ m^−^^3^, and excellent ionic conductivity of 33.607 mS cm^−^^1^ after electrolyte infiltration. Unlike traditional flexible matrices that passively buffer volume changes in silicon anodes, our binder actively responds to localized stress through microphase mediated deformation, enabling real–time redistribution of strain at critical interfaces via mechanochemical coupling mechanisms. Electrochemical testing confirmed that the Si@LRA/HT–PCNCs/SA–Ba^2^^+^ electrode maintained a high reversible capacity of 1798.3 mAh g^−^^1^ after 300 cycles at 0.5 C and delivered superior rate capability up to 869.8 mAh g^−^^1^ at 3 C, far exceeding conventional sodium alginate–based systems (367.2 mAh g^−^^1^). Furthermore, the designed binder promotes uniform lithium fluoride deposition at the interface to form a stable SEI layer for prolonged cycling durability. In summary, this bioinspired strategy leverages both mechanophore enabled adaptability and microphase network synergy for intelligent regulation of electrode mechanics and interface chemistry, offering new design principles beyond simple matrix stabilization with significant promise for next‐generation high‐energy density lithium‐ion batteries.

## Author Contributions

L.T. led writing, methodology, supervision, validation, project administration, and funding acquisition. L.Z. contributed to manuscript writing, experiments, formal analysis, and visualization. F.L. focused on experiments and formal analysis. H.L. and Z.C. conducted investigations. L.Q., Y.S., and Y.W. performed experiments. H.L., B.L., J.L., X.Y., J.T., and Z.W. supported validation and methodology. B.H. and X.Y. oversaw investigations and project administration.

## Funding

National Natural Science Foundation of China (Grant No. 32571993, Grant No. 32301539), Research Innovation Special Fund Project of Fujian Agriculture and Forestry University (Grant No. KFB24006).

## Conflicts of Interest

The authors declare no conflicts of interest.

## Supporting information




**Supporting File 1**: advs74493‐sup‐0001‐SuppMat.docx.

**Supporting File 2**: advs74493‐sup‐0002‐Video S1.mp4.

**Supporting File 3**: advs74493‐sup‐0003‐Video S2.mp4.

## Data Availability

The data that support the findings of this study are available from the corresponding author upon reasonable request.
