# Supplementary material for: Bioinspired Microphase‐Engineered Binders for Silicon Anodes
Source: Adv Sci (Weinh). 2026 Feb 21;13(25):e22802. doi: 10.1002/advs.202522802 (PMC13137806; doi:10.1002/advs.202522802)
Supplement: Supplementary file 1 — Supporting File 1: advs74493‐sup‐0001‐SuppMat.docx. [file ADVS-13-e22802-s001.docx]

Supporting Information

**Bioinspired Microphase-Engineered Binders with Adaptive Stress Dissipation for High Performance Silicon Anodes**

Lirong Tang ^*^, Lan Zhao ^*^, Zhiyi Cao, Fengcai Lin, Biao Huang, Haijun Li, Lingling Qian, Yingshan Shi, Yaohang Weng, Xuan Yang, Hanyang Liu, Beili Lu, Jianhua Lv, Xinda You, Jiayu Tao, Zhenwei Wu

This file includes:

**Experimental Section:**

Reagents

Synthesis of lipoic acid–rosin acrylate (LRA)

Synthesis of HT–PCNCs

Synthesis of LRA/HT–PCNCs/SA–Ba²⁺

Electrode preparation and battery assembly

**Figures:**

Fig. S1 FTIR of binders crosslinked with different metal ions.

Fig. S2 XPS C 1s of small molecule rosin–based polymer and binders.

Fig. S3 TG results of binders.

Fig. S4 SEM image of binders crosslinking structures with different metal ions.

Fig. S5 Rheological behavior of small molecule rosin–based polymers.

Fig. S6 Strain amplitude dynamic alternate sweep of LA.

Fig. S7 Storage modulus of dynamic frequency sweep.

Fig. S8 Loss modulus of dynamic frequency sweep.

Fig. S9 Storage modulus of dynamic stress sweep.

Fig. S10 Loss modulus of dynamic stress sweep.

Fig. S11 Strain amplitude dynamic alternate sweep of SA–Ba²⁺.

Fig. S12 Tensile properties of binders crosslinked with different metal ions.

Fig. S13 180 ° tear properties.

Fig. S14 Electrolyte infiltration of the binders.

Fig. S15 Electrolyte infiltration of other metal crosslinkers at 0 s.

Fig. S16 Swelling properties of binders.

Fig. S17 Comparison of other metal ion–bridged binders.

Fig. S18 Cycling performances of full cells at 0.5 C.

Fig. S19 CV curve of Si@LRA/HT–PCNCs/SA–Ba²⁺ electrode at 0.2 mV/s.

Fig. S20 Ionic conductivity of other metal ion crosslinked binders before and after electrolyte immersion.

Fig. S21 CV curves of Si@SA.

Fig. S22 LSV curves of the binders .

Fig. S23 Top view SEM images of electrodes before cycling.

Fig. S24 Cross–sectional SEM images of electrodes before cycling.

Fig. S25 Cross–sectional SEM images of electrodes after 300 cycles.

Fig. S26 AFM 3D images of Si@SA electrode before cycling.

Fig. S27 AFM 3D images of Si@SA electrode after cycling.

Fig. S28 AFM 3D images of Si@HT–PCNCs/SA electrode before cycling.

Fig. S29 AFM 3D images of Si@HT–PCNCs/SA electrode after cycling.

Fig. S30 HRTEM images of the Si@LRA/HT–PCNCs/SA–Ba²⁺ electrode at 10 nm, where the insets show the corresponding simplified inverse fast Fourier transform (IFFT) images of different regions.

Fig. S31 Comparison of the rate capability with recently reported Si anode binders.

Fig. S32 Comparison of the cycle performance with recently reported Si anode binders.

Table S1 Comparison of mechanical robustness and conductivities

**Experimental Section**

*Methods and Materials*

*Reagents**:* DL–lipoic acid (LA), sodium alginate (SA), ferric chloride hexahydrate (FeCl₃·6H₂O, 99%), zirconium tetrachloride (ZrCl₄, ≥98%), anhydrous lithium hydroxide (LiOH), and phosphoric acid (85 wt%) were purchased from Aladdin Biochemical Technology Co., Ltd. Barium chloride dihydrate (BaCl₂·2H₂O, ≥99.5%), zinc chloride (ZnCl₂, ≥98%), nickel chloride hexahydrate (NiCl₂·6H₂O, ≥98.0%), ferrous chloride tetrahydrate (FeCl₂·4H₂O, ≥98.0%), copper(II) chloride dihydrate (CuCl₂·2H₂O, ≥99.0%), urea (≥99.0%), acetone, and ethanol (≥99.7%) were obtained from Sinopharm Chemical Reagent Co., Ltd. Microcrystalline cellulose (MCC) was supplied by Beijing Solarbio Science & Technology Co., Ltd. Tween 80 was purchased from Macklin Biochemical Technology Co., Ltd., and acrylic rosin was provided by Arakawa Chemical Industries, Ltd. Silicon nanoparticles (SiNPs, ≥99%, average particle size ~30 nm) were obtained from ST–NANO Material Technology Co., Ltd. Multi-walled carbon nanotubes (MWCNTs, ≥90%) were acquired from Nanocyl SA. The lithium-ion battery electrolyte (LB–276) was supplied by DodoChem Technology Co., Ltd., and lithium metal was provided by Tianjin Zhongneng Lithium Industry Co., Ltd. The porous polyethylene separator (Celgard 2500) was purchased from Celgard, USA. Commercial LiFePO₄ and LiCoO₂ cathode materials were obtained from Guangdong Zhuguang New Energy Technology Co., Ltd.

*Synthesis of lipoic acid–rosin acrylate (LRA)*

Lipoic acid–rosin acrylate (LRA) was synthesized via a straightforward one-step method. Briefly, 0.5 g of lipoic acid was dissolved in 0.7 mol L⁻¹ LiOH solution under stirring and heating for 5 minutes. Subsequently, 0.21 g, 0.42 g, 0.63 g, 0.84 g, and 1.68 g of acrylated rosin, corresponding to molar ratios of 0.5:1, 0.5:1, 0.75:1, 1:1, and 2:1, respectively, were each dissolved in a minimal amount of acetone and slowly added to the reaction vessel. The mixture was continuously stirred and heated for 15 minutes until the acetone had completely evaporated, promoting thorough crosslinking and yielding a milky-yellow product. Thereafter, 0.05 mL of Tween 80 was added dropwise. After complete mixing, the solution was allowed to stand and cool to room temperature. The final solid content was approximately 40 wt%. The synthesis of poly(lipoic acid) followed the same procedure, except that acrylated rosin (AR) was omitted.

*Synthesis of HT–PCNCs*

A suspension of 4 g of PCNCs was subjected to hydrothermal treatment at 145 °C for 15 hours. The resulting product was washed thoroughly with deionized water until the filtrate reached neutral pH, followed by vacuum drying and sieving through a 300-mesh screen to obtain hinged-tethering phosphorylated cellulose nanocrystals (HT–PCNCs).

*Synthesis of LRA/HT–PCNCs/SA–Ba²⁺*

To prepare the LRA/HT–PCNCs/SA–Ba²⁺ composite, 0.02 mmol of BaCl₂·2H₂O, 100 mg of HT–PCNCs, and 1 g of SA were dissolved in 20 mL of deionized water and stirred for 3 hours. Subsequently, 18 g of the resulting mixture was combined with 0.25 g of LRA and stirred at 80 °C for 30 minutes to ensure uniform mixing. The final product was designated as LRA/HT–PCNCs/SA–Ba²⁺. For comparison, other metal-ion-crosslinked composites (Ni²⁺, Zn²⁺, Cu²⁺, Ba²⁺, Fe²⁺, Fe³⁺, and Zr⁴⁺) were synthesized using the same procedure and labeled as LRA/HT–PCNCs/SA–M^n⁺^. The SA–Ba²⁺ and HT–PCNCs/SA control samples were prepared following the same protocol, excluding the addition of the respective other components.

*Electrode preparation and battery assembly*

To fabricate the Si electrode, a slurry was prepared by mixing Si active material, conductive agent, and binder in a mass ratio of 70:10:20 using deionized water as the dispersing medium. The binders used included LRA/HT–PCNCs/SA–Ba²⁺, HT–PCNCs/SA, and SA–Ba²⁺, with SA serving as the control. The resulting electrodes were designated as Si@LRA/HT–PCNCs/SA–Ba²⁺, Si@HT–PCNCs/SA, Si@SA–Ba²⁺, and Si@SA, respectively. The homogeneous slurry was uniformly coated onto a copper foil current collector and dried in a vacuum oven at 80 °C for 12 hours to remove residual moisture. The dried electrodes were then punched into 12 mm diameter discs with an active material loading of 0.3–0.5 mg/cm². The LiFePO_4_ and LiCoO_2_ cathode slurries were prepared by mixing the active materials, polyvinylidene fluoride (PVDF), and Super P with a mass ratio of 90:5:5 in N-methyl-2-pyrrolidone (NMP). The resultant slurry was blade-coated onto aluminum foil and subsequently dried at 80 °C for 12 h under vacuum.

CR2025 coin cells incorporating the aforementioned battery binders were assembled in an argon-filled glove box, where moisture and oxygen levels were maintained below 0.1 ppm. The electrolyte consisted of 1.0 mol/L LiPF₆ dissolved in a solvent mixture of ethylene carbonate (EC), diethyl carbonate (DEC), and ethyl methyl carbonate (EMC) in a 1:1:1 volume ratio, with the addition of 10 wt% fluoroethylene carbonate (FEC) as an electrolyte additive. A Celgard 2500 membrane was used as the separator, and lithium foil served as the counter electrode.

*Structural and Chemical Characterization*

FTIR spectroscopy (VERTEX 70, Bruker, Germany) was used in the transmission mode (400–4000 cm⁻¹) to identify functional groups and assess molecular interactions. The PIB index was used to quantify the chelation between SA and metal ions.

*PIB** = ∆*v*(COONa)/∆*v*(COOMe).

∆*v*(COONa) is defined as the difference between the asymmetric and symmetric stretching peaks of SA carboxylate groups in the FTIR spectrum. ∆*v*(COOMe) is the corresponding shift observed after metal crosslinking.

^1^H NMR (Avance NEO 400 MHz, Bruker, Germany) was used to assess chemical shift caused by local structural changes. XRD (MAXima XRD–7000, Shimadzu, Japan) with Cu K_α_ radiation was used to analyze crystallinity and crosslinking, at a scan rate of 5 ° min^-^¹ over 5–80° or 5–50°. Data were interpreted using the Bragg’s equation:

*nλ* = 2*d*sin*θ*,

where *λ* is the X–ray wavelength, *θ* is the incident angle, *d* is the interplanar spacing, and *n* is the diffraction order. The surface morphology was characterized using field–emission SEM (Nova NanoSEM 230, FEI, USA), TEM (JEM–F200, JEOL, Japan) and AFM (Dimension Edge, Bruker, Germany) to evaluate the microstructure and interfacial bonding of the binder before and after modification.

Elemental composition and chemical states were further analyzed by XPS (AXIS UltraDLD, Kratos, UK).

*Nonelectrochemical Characterization*

The binder performance was systematically evaluated for thermal and mechanical reliability. The thermal stability was assessed using a simultaneous thermal analysis (STA449F3, Netzsch, Germany), with heating to 800 °C at 10 K min^-^¹ under an inert gas to confirm structural integrity.

Rheological properties, crucial for a uniform electrode coating, were measured with a rotational rheometer (MARS III, Thermo Fisher, USA), analyzing viscosity, storage modulus, and loss modulus.

Mechanical strength and adhesion were tested with a universal testing machine (WDH–50N, Guanteng, China) through tensile and 180° peel tests. Local mechanical properties were characterized via nanoindentation (Nano Indenter G200, Keysight, USA) at a maximum load of 100 μN to assess hardness and modulus.

Swelling tests were repeated at least three times to characterize structural changes of the binder in the electrolyte. The swelling ratio (SR) was calculated as *SR* = $\frac{\text{m}_{\text{1 }}\text{- }\text{m}_{\text{0}}}{\text{m}_{\text{0}}}\text{,}$ where *m*₀ is the initial mass of the film and *m*₁ is the mass after soaking in the electrolyte for 72 h and blotting excess liquid from the surface. Contact angle measurements (DSA 30, KRÜSS, Germany) were used to assess wettability and interfacial compatibility between the electrolyte, binder film, and electrode, ensuring an effective electrolyte penetration and stable electrode–electrolyte contact.

*Electrochemical Characterization*

Ionic conductivity, a key parameter for binder evaluation, was measured at least three times using an inductance–capacitance–resistance (LCR) meter (VC4092A, Shengli, China) with the binder films cut into dimensions of 20 mm × 5 mm × 0.1 mm. The coin cell cycling performance was tested via constant current charge–discharge in the range of 0.01–1.5 V using a LAND battery tester (CT3002A, China). EIS was performed using an electrochemical workstation (CHI600E, Chenhua, China) across 0.01–100 kHz. LSV tests was scanned under the speed of 1 mV s^-^¹ (0.01–7 V). CV at various scan rates was used to evaluate lithium–ion transport, calculated as

$\text{I}_{\text{p}}\text{=2.69×}\text{10}^{\text{5}}\text{ }\text{n}^{\text{1.5}}\text{A}\text{C}_{\text{Li}^{\text{+}}}\text{ν}^{\text{0.5}}{\text{D}_{\text{Li}^{\text{+}}}}^{\text{0.5}}\text{,}$

where *I*_p_ is the peak current, *n* is the number of transferred electrons, *A* is the working electrode area (1.13 cm²), *C*_Li⁺_ is the lithium-ion concentration in the electrolyte (0.001 mol cm^-^³), *D*_Li⁺_ is the lithium-ion diffusion coefficient, and *v* is the scan rate, set at 0.2, 0.4, 0.6, 0.8, and 1.0 mV s^-^¹. To further evaluate ion transport, GITT was employed on half-cells using the LAND system over a voltage range of 0.01–1.5 V. Cells were alternately charged/discharged at 0.2 C for 10 min followed by a 30 min relaxation.

According to the Fick’s second law, the lithium-ion diffusion coefficient was calculated using the equation.

$\text{D}_{\text{Li}^{\text{+}}}$= $\frac{\text{4}}{\text{πt}}\left( \frac{\text{m}_{\text{A}}\text{V}_{\text{m}}}{\text{M}_{\text{A}}\text{S}} \right)^{\text{2}}\left( \frac{\text{Δ}\text{E}_{\text{p}}}{\text{Δ}\text{E}_{\text{cc}}} \right)^{\text{2}}\text{,}$

where *t* is the duration of the charge/discharge step, *m*_A_, *V*_m_, and *M*_A_ are the mass, molar volume, and molar mass of the active material, respectively, *S* is the effective reaction area, Δ*E_p_* is the voltage change during the pulse, and Δ*E_cc_* is the voltage change during constant-current charge/discharge.

*Calculation method*

All first-principles calculations are performed using the plane-wave projector-augmented wave method, as implemented in the Vienna ab initio simulation package (VASP). The Perdew−Burke−Ernzerhof (PBE) form of generalized gradient approximation (GGA) is chosen as the exchange−correlation potential. Grimme’s DFT-D3(BJ) method was used to describe the vdW interaction. The energy cutoff of 520 eV was used for structural relaxation. k-Spacing was set to 0.4 for all structures to allow the smallest spacing between k-points in the unit of 0.4 Å^–1^. The conjugated gradient method is utilized to optimize the geometry with the convergence threshold of 10-5 eV in energy and 0.02 eV/Å in force, respectively. The vacuum thickness over 20Å was used to reduce the periodic interactions.

The adsorption energy of molecule/ion A on Si (110) surface was defined as

E_ab_ = E_total_–E_A_–E_Si_ (110)

Where E_total_ is the energy of the Si (110) surface after adsorption of A, E_A_ is the energy of single A molecule/ion, and E_Si_ (110) is the energy of Si (110) surface.

All quantum chemical calculations in this work were performed with B3LYP/6–31+G (d,p) level using the framework of Gaussian16 program package.

*Statistical Analysis*

This study focuses on trend-based analysis of electrochemical and materials characterization results. Quantitative data from swelling ratio and ionic conductivity tests were obtained from at least three independent samples (n ≥ 3) and are presented as mean values with standard deviation, as shown by the error bars in the corresponding figures. Other results, including electrochemical performance (cycling, GITT, EIS, CV), spectroscopy (FTIR, XPS, NMR), microscopy (SEM, AFM, TEM), thermal, and mechanical analyses, were interpreted based on consistent and representative observations from independently conducted experiments. Data processing and visualization were performed using OriginPro (Version 2025b, OriginLab) and Microsoft Excel. No statistical hypothesis testing was applied.

**Figures**

**
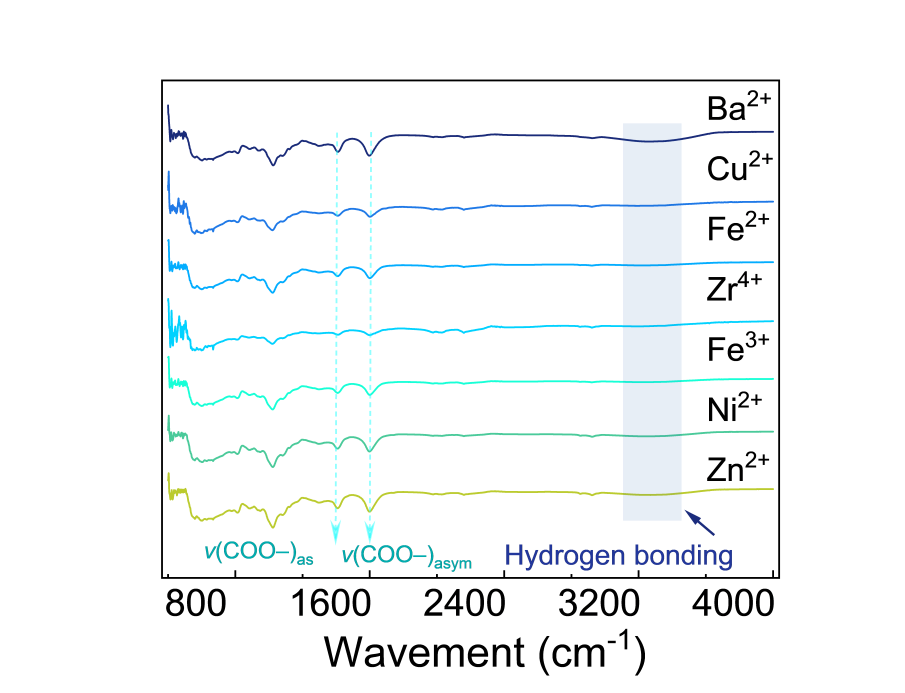
**

**Fig. S1** FTIR of binders crosslinked with different metal ions.


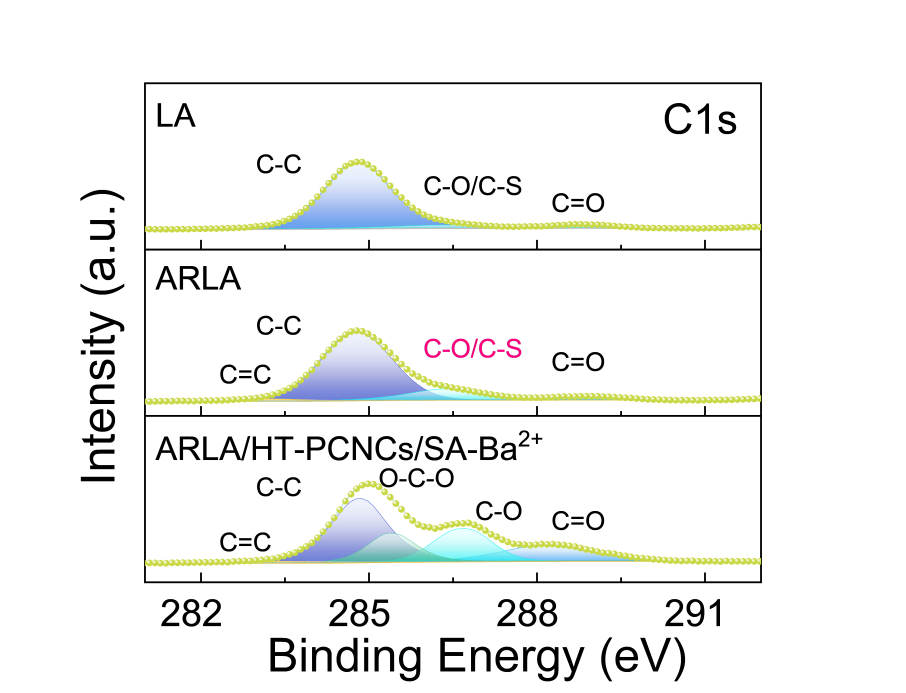


**Fig. S2** XPS C 1s of small molecule rosin–based polymer and binders.

**Fig. S3** TG results of binders.


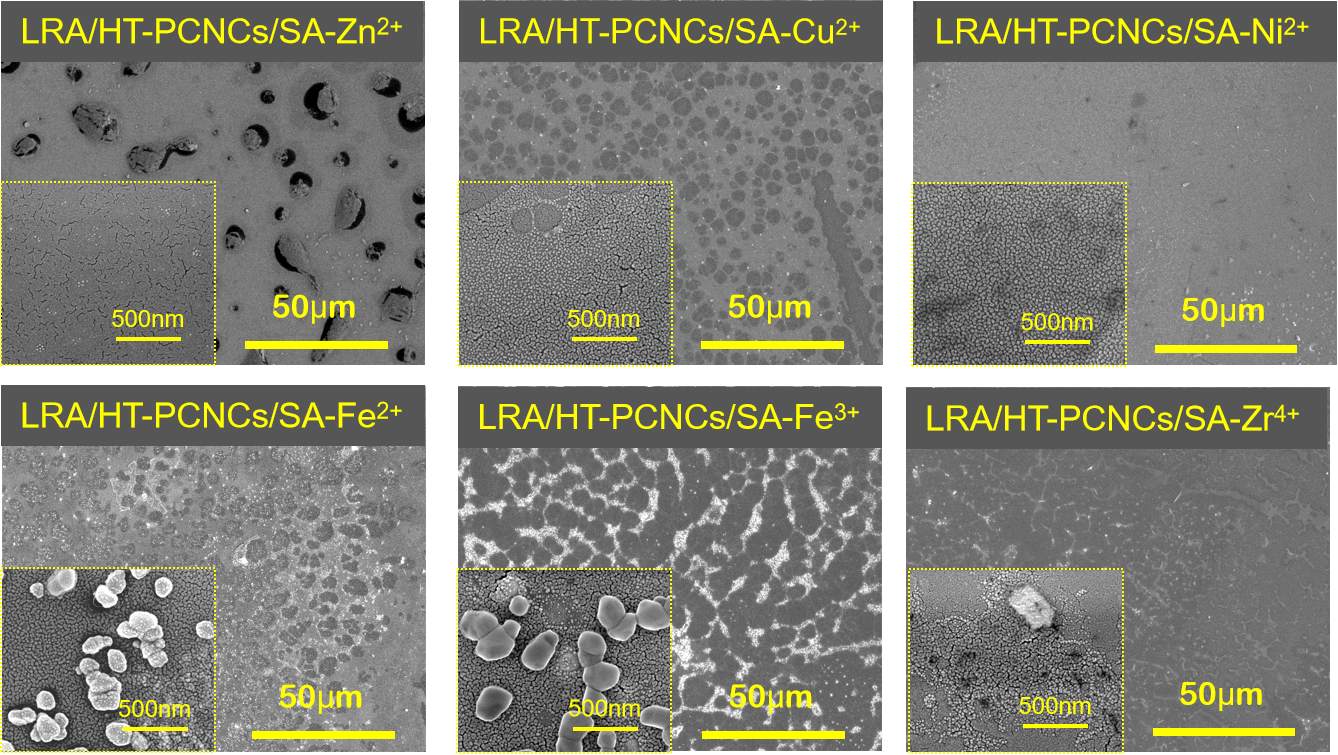


**Fig. S4** SEM image of binders crosslinking structures with different metal ions.

**Fig. S5** Rheological behavior of small molecule rosin–based polymers.

**Fig. S6** Strain amplitude dynamic alternate sweep of LA.

**Fig. S7** Storage modulus of dynamic frequency sweep.

**Fig. S8** Loss modulus of dynamic frequency sweep.

**Fig. S9** Storage modulus of dynamic stress sweep.

**Fig. S10** Loss modulus of dynamic stress sweep.

**Fig. S11** Strain amplitude dynamic alternate sweep of SA–Ba²⁺.

**Fig. S12** Tensile properties of binders crosslinked with different metal ions.

**Fig. S13** 180 ° tear properties.

**Fig. S14** Electrolyte infiltration of the binders.


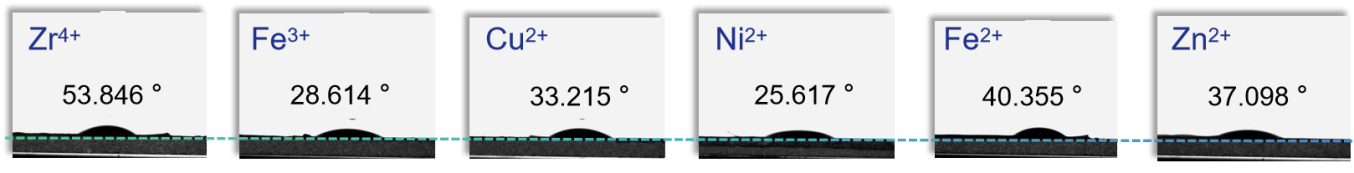


**Fig. S15** Electrolyte infiltration of other metal crosslinkers at 0 s.

**Fig. S16** Swelling properties of binders.

**Fig. S17** Comparison of other metal ion–bridged binders.


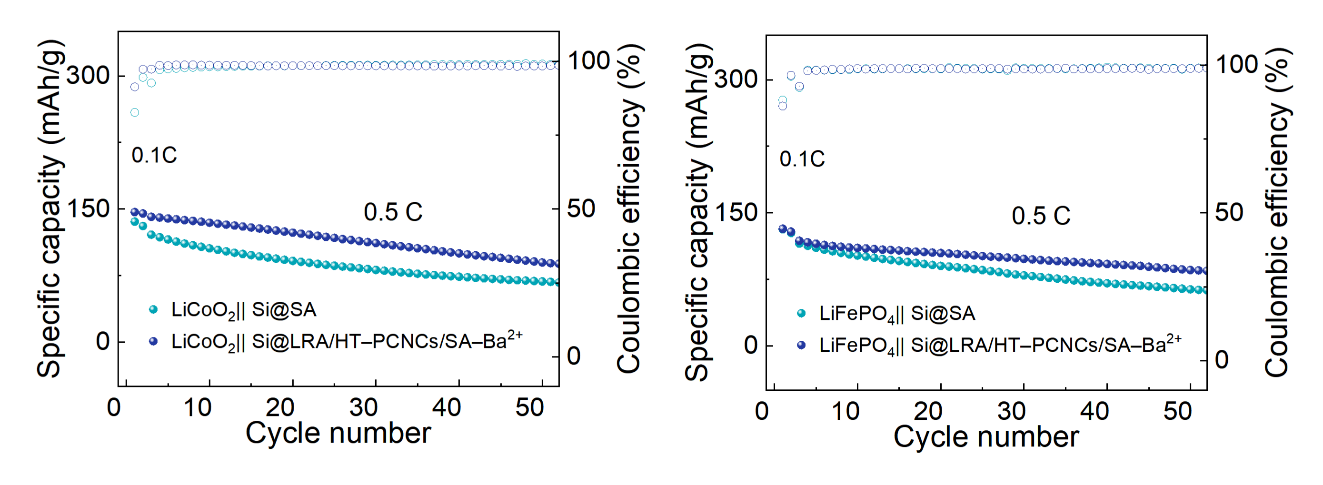
**Fig.S18** Cycling performances of full cells at 0.5 C.

**Fig. S19** CV curve of Si@LRA/HT–PCNCs/SA–Ba^²⁺^ electrode at 0.2 mV/s.

**Fig. S20** Ionic conductivity of other metal ion crosslinked binders before and after electrolyte immersion.

**Fig. S21** CV curves of Si@SA.


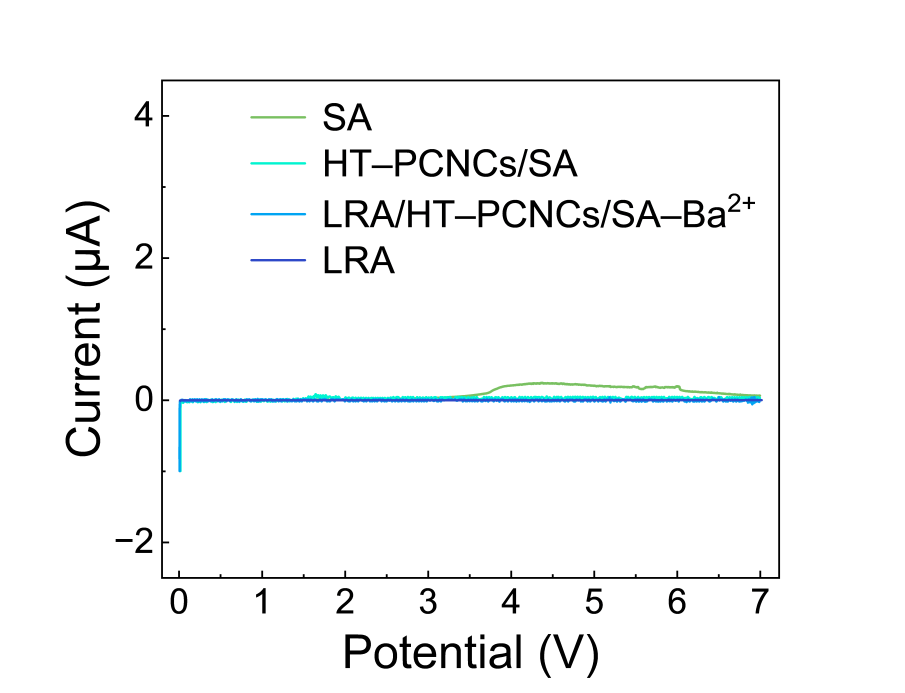


**Fig. S22** LSV curves of the binders.


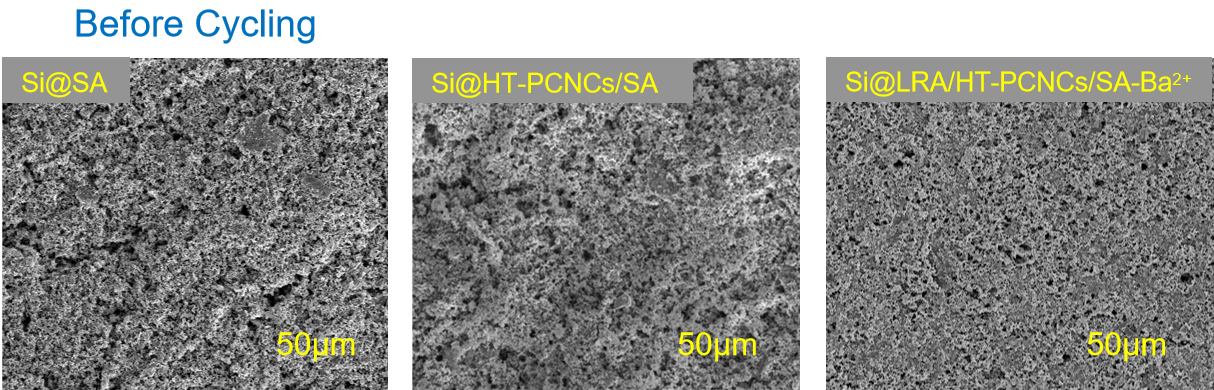


**Fig. S23** Top view SEM images of electrodes before cycling.


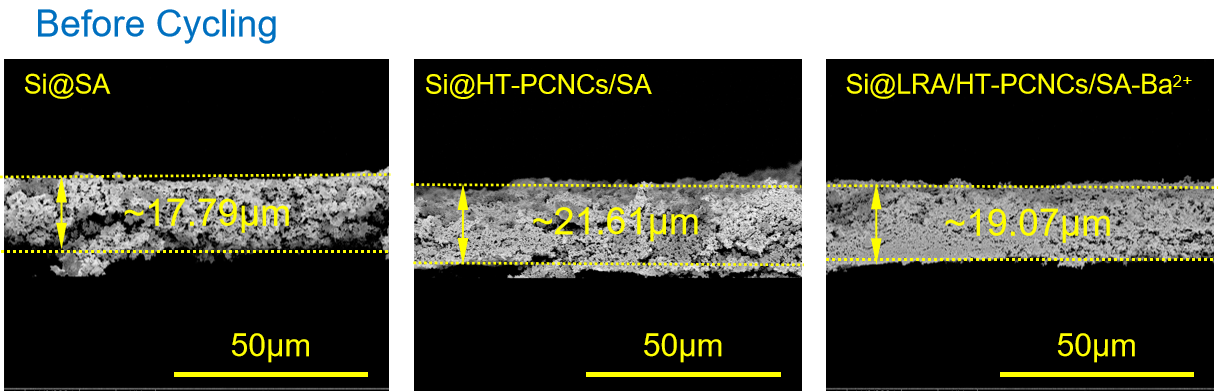


**Fig. S24** Cross–sectional SEM images of electrodes before cycling.


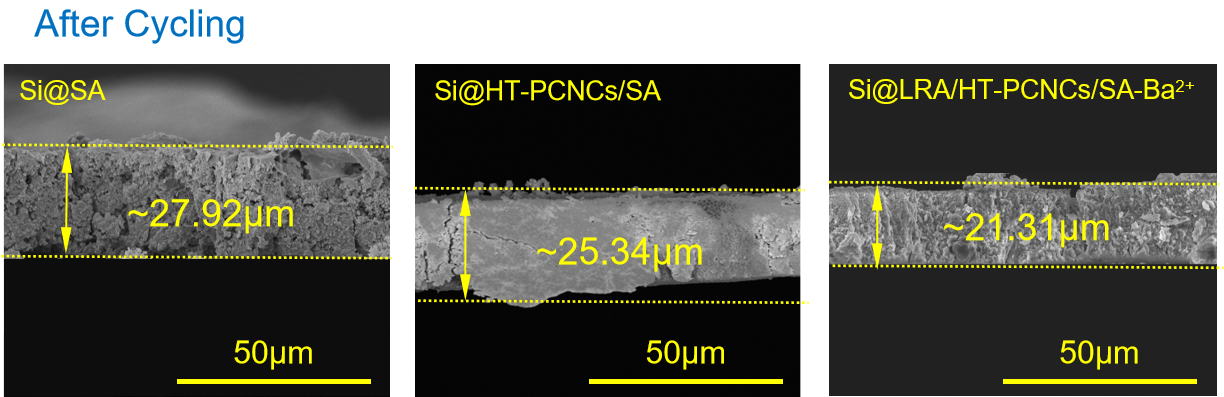


**Fig. S25** Cross–sectional SEM images of electrodes after 300 cycles.


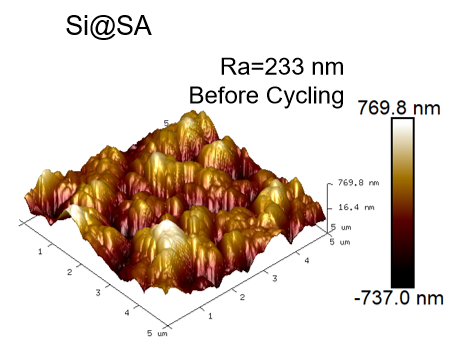


**Fig. S26** AFM 3D images of Si@SA electrode before cycling.


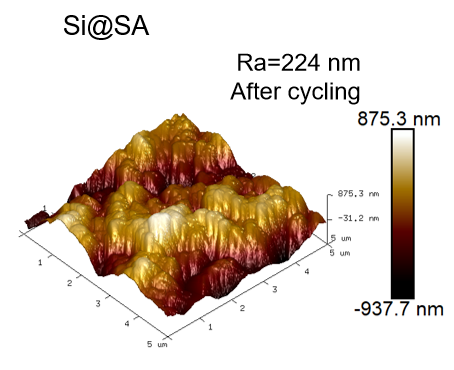


**Fig. S27** AFM 3D images of Si@SA electrode after cycling.


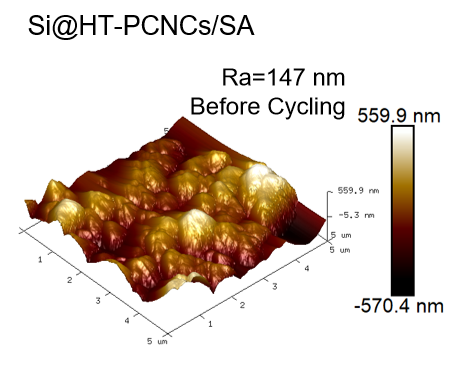


**Fig. S28** AFM 3D images of Si@HT–PCNCs/SA electrode before cycling.


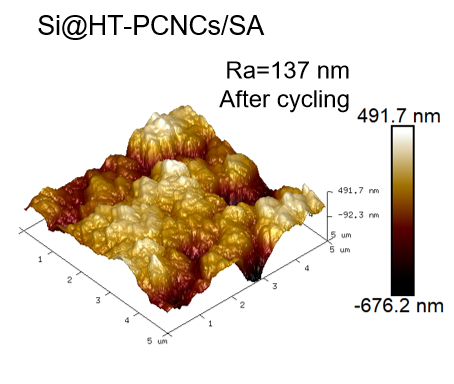


**Fig. S29** AFM 3D images of Si@HT–PCNCs/SA electrode after cycling.


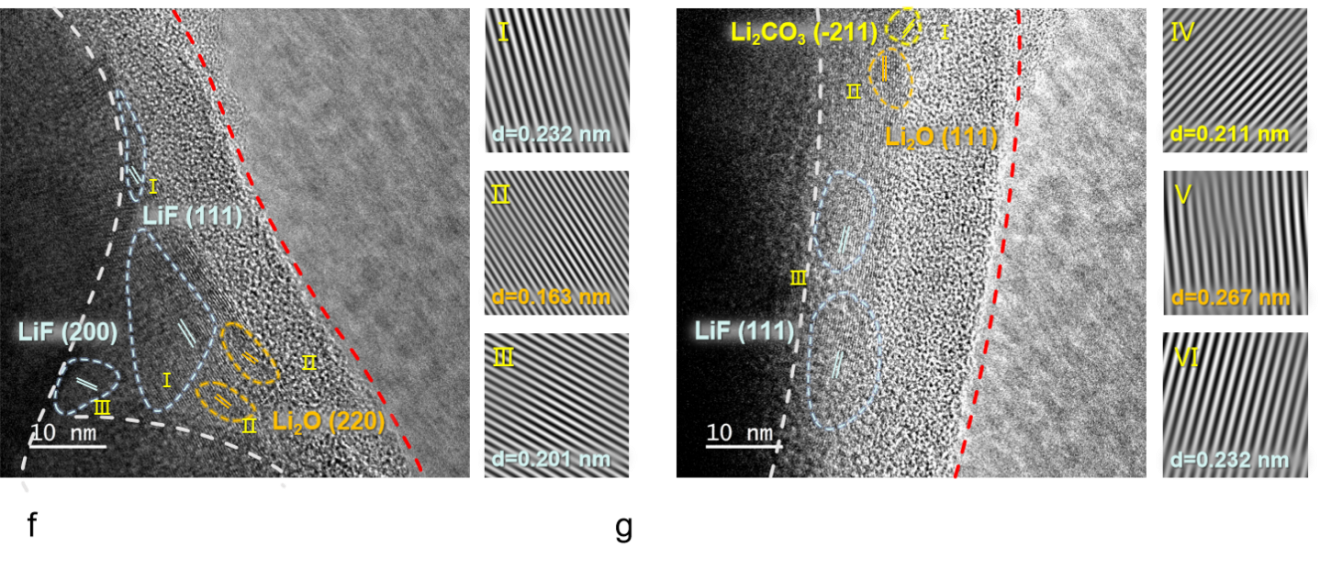


**Fig. S30** HRTEM images of the Si@LRA/HT–PCNCs/SA–Ba²⁺ electrode at 10 nm, where the insets show the corresponding simplified inverse fast Fourier transform (IFFT) images of different regions.


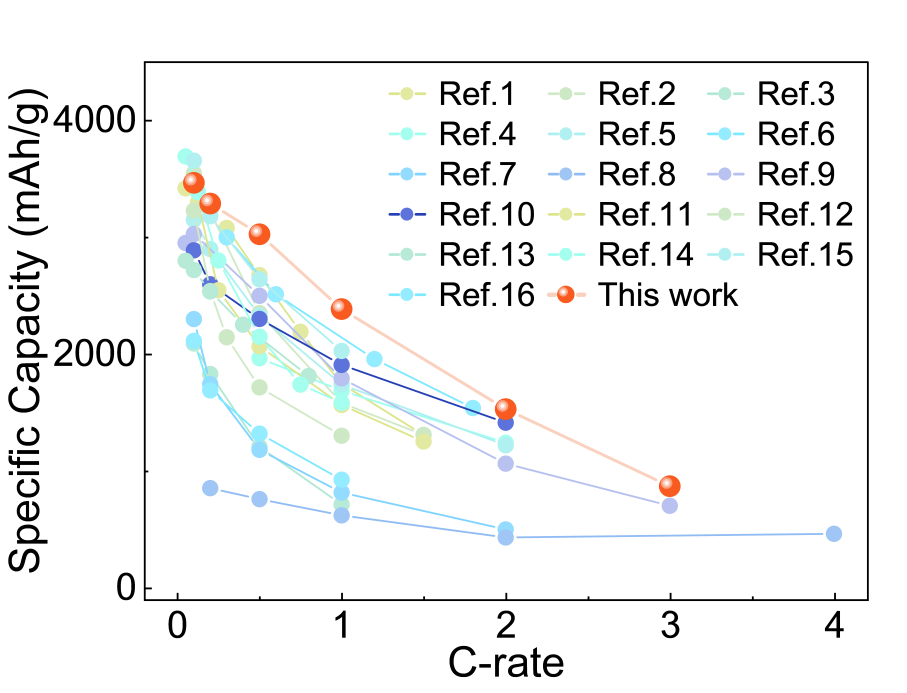


**Fig. S31** Comparison of the rate capability with recently reported Si anode binders.^[1–16]^


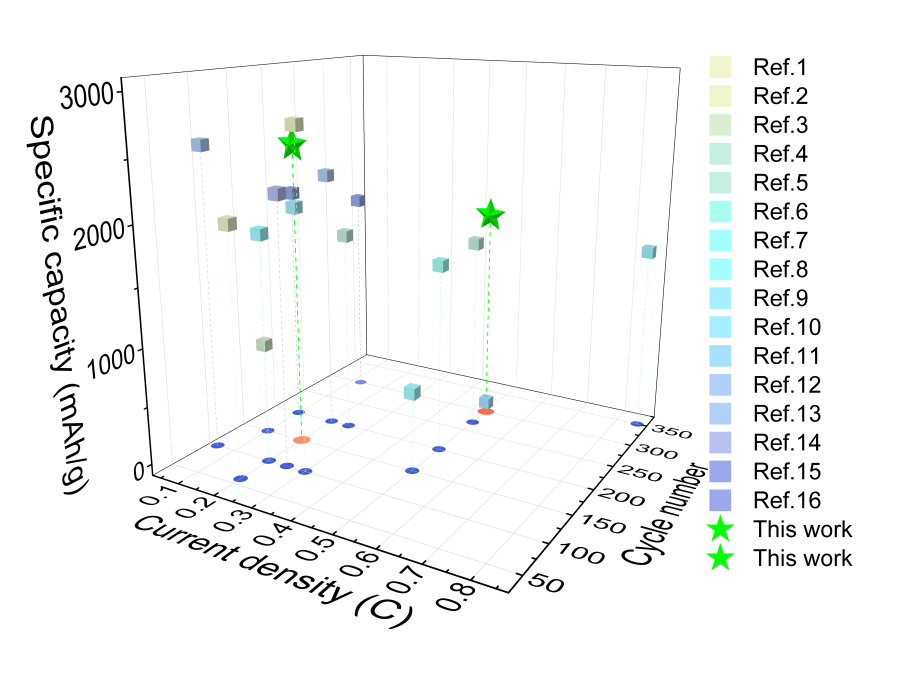


**Fig. S32** Comparison of the cycle performance with recently reported Si anode binders.^[1–16]^

**Table S1** Comparison of mechanical robustness and conductivities

| Category | Material (Binder System) | Maximum stress/ MPa | Maximum strain/ % | Ion conduction/  mS cm⁻¹ | Electronic transport/ mS cm⁻¹ |
| --- | --- | --- | --- | --- | --- |
| Precursors | LA (Lipoic Acid) | 0.38×10⁻³ | 1233.08 | 1.743 ± 0.054 | (0.771 ± 0.116) ×10⁻³ |
|  | LRA (Pre-polymer) | 4.89×10⁻³ | 4154.48 | 2.989 ± 0.260 | (3.730 ± 1.230) ×10⁻³ |
| Controls | SA | 45.32 | 3.78 | 6.518 ± 1.344 | 0.026 ± 0.001 |
|  | HT–PCNCs/SA | 77.16 | 3.50 | 18.268 ± 8.538 | 0.054 ± 0.021 |
|  | SA–Ba²⁺ | 50.41 | 0.67 | 20.190 ± 4.187 | 0.046 ± 0.027 |
| **Optimized Work** | **LRA/HT–PCNCs/SA–Ba²⁺** | **308.52** | **16.01** | **33.607 ± 0.620** | **0.196 ± 0.006** |
| Cation Screening | LRA/HT–PCNCs/SA–Fe²⁺ | 125.23 | 6.76 | 26.202 ± 6.116 | 0.057 ± 0.011 |
| (+2 Valence) | LRA/HT–PCNCs/SA–Zn²⁺ | 182.33 | 2.19 | 26.549 ± 5.135 | 0.077 ± 0.005 |
|  | LRA/HT–PCNCs/SA–Ni²⁺ | 115.44 | 7.54 | 28.243 ± 2.648 | 0.078 ± 0.027 |
|  | LRA/HT–PCNCs/SA–Cu²⁺ | 92.93 | 2.18 | 29.200 ± 1.195 | 0.106 ± 0.015 |
| (High Valence) | LRA/HT–PCNCs/SA–Fe³⁺ | 147.37 | 3.945 | 15.833 ± 1.563 | 0.041 ± 0.014 |
|  | LRA/HT–PCNCs/SA–Zr⁴⁺ | 77.98 | 1.30 | 23.412 ± 1.418 | 0.033 ± 0.004 |

**Reference**

[1] X. Jiao, J. Yin, X. Xu, J. Wang, Y. Liu, S. Xiong, Q. Zhang, J. Song, “Highly Energy-Dissipative, Fast Self-Healing Binder for Stable Si Anode in Lithium-Ion Batteries,” *Advanced Functional Materials* 30 (2021): 2005699. <https://doi.org/10.1002/adfm.202005699>

[2] H. Chen, Z. Wu, Z. Su, S. Chen, C. Yan, M. Al-Mamun, Y. Tang, S. Zhang, “A Mechanically Robust Self-Healing Binder for Silicon Anode in Lithium Ion Batteries,” *Nano Energy* 81 (2021): 105654. <https://doi.org/10.1016/j.nanoen.2020.105654>

[3] W.J. Kim, J.G. Kang, D.W. Kim, “Blood Clot-Inspired Viscoelastic Fibrin Gel: New Aqueous Binder for Silicon Anodes in Lithium Ion Batteries,” *Energy Storage Materials* 45 (2022): 730-740. <https://doi.org/10.1016/j.ensm.2021.12.034>

[4] Z. Li, G. Wu, Y. Yang, Z. Wan, X. Zeng, L. Yan, S. Wu, M. Ling, C. Liang, K.N. Hui, Z. Lin, “An Ion‐Conductive Grafted Polymeric Binder with Practical Loading for Silicon Anode with High Interfacial Stability in Lithium‐Ion Batteries,” *Advanced Energy Materials* 12, no. 29 (2022): 2201197. <https://doi.org/10.1002/aenm.202201197>

[5] D. Jeong, J. Yook, D.S. Kwon, J. Shim, J.C. Lee, “Interweaving Elastic and Hydrogen Bond‐Forming Polymers into Highly Tough and Stress‐Relaxable Binders for High‐Performance Silicon Anode in Lithium‐Ion Batteries,” *Advanced Science* 10, no. 31 (2023): 2302027. <https://doi.org/10.1002/advs.202302027>

[6] D. Zhang, Y. Ouyang, Y. Wang, L. Liu, H. Wang, J. Cui, M. Wang, N. Li, H. Zhao, S. Ding, “A Gradient-Distributed Binder with High Energy Dissipation for Stable Silicon Anode,” *Journal of Colloid and Interface Science* 673 (2024): 312-320. <https://doi.org/10.1016/j.jcis.2024.06.086>

[7] N.J. Kong, M.S. Kim, J.H. Park, J. Kim, J. Jin, H.W. Lee, S.J. Kang, “Promoting Homogeneous Lithiation of Silicon Anodes via the Application of Bifunctional PEDOT: PSS/PEG Composite Binders,” *Energy Storage Materials* 64 (2024): 103074. <https://doi.org/10.1016/j.ensm.2023.103074>

[8] J. Kim, J. Choi, K. Park, S. Kim, K.W. Nam, K. Char, “Host–Guest Interlocked Complex Binder for Silicon–Graphite Composite Electrodes in Lithium-Ion Batteries,” *Advanced Energy Materials* 12 (2022): 2103718. <https://doi.org/10.1002/aenm.202103718>

[9] X. Lin, D. Ma, Z. Zhu, S. Wang, H. Liu, X. Xu, Z. Li, “Spider Web-Inspired Structural Design for an Energy-Dissipating Polymer Binder Enabling Stabilized Silicon Anodes,” *Journal of Energy Chemistry* 109 (2025): 870-878. <https://doi.org/10.1016/j.jechem.2025.06.020>

[10] D. Cheng, F. Song, Y. Zeng, D. Qin, Z. Ma, P. Zheng, G. Zhang, C. Wang, Y. Qian, “Dynamic Self‐Adaption Supramolecular Binder for Silicon Anodes: Anhydride Activation Enabling Practical Lithium‐Ion Battery,” *Advanced Functional Materials* (2025): 2507041. <https://doi.org/10.1002/adfm.202507041>

[11] S. Wu, L. He, Y. Lu, J. Zheng, L. Li, X. Geng, C. Sun, H. Zhao, G. Jiang, F. Di, B. An, “Volumetric Stress Managements on Silicon Anode of Lithium‐Ion Batteries by a Self‐Adaptable Binder,” *Energy & Environmental Materials* 8, no. 3 (2025): e12859. <https://doi.org/10.1002/eem2.12859>

[12] J. Shen, S. Zhang, H. Wang, R. Wang, Y. Hu, Y. Mao, R. Wang, H. Zhang, Y. Du, Y. Fan, Y. Zhou, Z. Guo, B. Wang, “Unlocking the Potential of Silicon Anodes in Lithium-Ion Batteries: A Claw-Inspired Binder with Synergistic Interface Bonding,” *eScience* 4, no. 3 (2024): 100207. <https://doi.org/10.1016/j.esci.2023.100207>

[13] B. Chen, D. Xu, S. Chai, Z. Chang, A. Pan, “Enhanced Silicon Anodes with Robust SEI Formation Enabled by Functional Conductive Binder,” *Advanced Functional Materials* 34, no. 34 (2024): 2401794. <https://doi.org/10.1002/adfm.202401794>

[14] W. Zhou, R. Zhang, S. Yu, Z. Peng, C. Zuo, W. Yang, Y. Li, M. Wei, “High‐Branched Natural Polysaccharide Flaxseed Gum Binder for Silicon‐Based Lithium‐Ion Batteries with High Capacity,” *Small* 20, no. 36 (2024): 2403048. <https://doi.org/10.1002/smll.202403048>

[15] Y. Wang, X. Yang, Y. Yuan, Z. Wang, H. Zhang, X. Li, “N‐Rich Solid Electrolyte Interface Constructed in Situ via a Binder Strategy for Highly Stable Silicon Anode,” *Advanced Functional Materials* 33, no. 34 (2023): 2301716. <https://doi.org/10.1002/adfm.202301716>

[16] S. Dai, F. Huang, J. Yan, Y.Y. Sun, C. Chen, H. Li, “Construction of Protein‐Like Helical‐Entangled Structure in Lithium‐Ion Silicon Anode Binders via Helical Recombination and Hofmeister Effect,” *Advanced Science* 12, no. 20 (2025): 2412769. <https://doi.org/10.1002/advs.202412769>
